# Expression of antisense small RNAs in response to stress in *Pseudomonas aeruginosa*

**DOI:** 10.1186/1471-2164-15-783

**Published:** 2014-09-11

**Authors:** María Gómez-Lozano, Rasmus L Marvig, Monica VL Tulstrup, Søren Molin

**Affiliations:** Department of Systems Biology, Technical University of Denmark, Lyngby, Denmark; Novo Nordisk Foundation Center for Biosustainability, Technical University of Denmark, Hørsholm, Denmark

## Abstract

**Background:**

RNA sequencing technologies reveal that bacteria express RNA molecules other than mRNA, rRNA or tRNA. During the last years genome-wide bacterial transcriptomes have been shown to comprise intergenic RNA, antisense RNA, and untranslated regions, all capable of performing diverse regulatory functions.

**Results:**

In this study we used RNA-seq to identify 232 antisense RNAs (asRNAs) in the opportunistic pathogen *Pseudomonas aeruginosa* grown under 13 different conditions. The conditions studied include exponential and stationary growth as well as osmotic, oxidative and antibiotic stress. We found a significant overrepresentation of asRNAs that are transcribed opposite to genes involved in cell division and in cell wall, lipopolysaccharide (LPS), and capsule biosynthesis, most likely reflecting the conditions used in this study. A substantial number of asRNAs significantly changed their expression under osmotic, oxidative and antibiotic stress, suggesting that asRNAs may play regulatory roles during these conditions. We also made a comparison between the asRNAs detected in this study in *P. aeruginosa* PAO1 with the asRNAs detected in two previous studies in *P. aeruginosa* PA14, and found that the extent of overlap between the studies is very limited.

**Conclusions:**

RNA-seq experiments are revealing hundreds of novel transcripts in all bacterial genomes investigated. The comparison between independent studies that used RNA-seq to detect novel asRNAs in *P. aeruginosa* shows that the overlap between the results reported is very narrow. It is necessary to address how reproducibility of these kind of studies should be reported in order to avoid misleading conclusions when comparing data generated by non-identical methods.

**Electronic supplementary material:**

The online version of this article (doi:10.1186/1471-2164-15-783) contains supplementary material, which is available to authorized users.

## Background

During the last years an increasing number of bacterial transcriptomes have been examined with tiling arrays and RNA sequencing (RNA-Seq) technologies, revealing that a significant number of protein-coding genes are also transcribed from the reverse complementary strand in a wide range of bacterial species [1–8]. Overlapping transcription results in the generation of antisense RNAs (asRNA) with sizes ranging from tens to thousands of nucleotides (nt). Regulatory roles of asRNAs were first reported more than 30 years ago in *Escherichia coli,* where plasmid-encoded asRNAs were found to negatively regulate plasmid copy number [9–11]. Since then bacterial asRNAs were only occasionally detected and therefore considered to be rare, and as late as in 2007 only about thirty bacterial asRNAs had been identified (reviewed in [12]). However, due to the use of tiling array and especially RNA-Seq the number of reported asRNAs in bacterial transcriptomes is now rapidly growing. The characterization of the physiological function of individual asRNAs is however lagging behind. AsRNAs are known to affect the expression of the target gene by different mechanisms (reviewed in [13]). These include: (i) interference by convergent transcription, in which transcription from one promoter is suppressed by a second promoter located in the opposite strand, (ii) transcription attenuation, in which base pairing of the asRNA to the target mRNA causes premature transcription termination, (iii) changes in the target RNA stability, where the asRNA either promotes or blocks degradation or cleavage of the mRNA by ribonucleases, (iv) asRNAs can directly block ribosome binding, and (v) might indirectly impact ribosome binding (either positively or negatively) by affecting the target RNA structure. In addition, regulating the expression of the opposite gene is not the only function of certain asRNAs. Some asRNAs encode small proteins [14], and some have the potential to act on multiple targets in *trans* [15–19].

In this work, we used RNA-Seq to identify asRNAs in the human pathogen *P. aeruginosa*, which can cause severe infections, particularly in immunocompromised and cystic fibrosis (CF) patients. The CF lung is an osmotically stressful environment, due to the viscous, dehydrated mucus, cellular debris and electrolytes competing for a limited supply of water [20, 21]. A variety of studies have shown that *P. aeruginosa* encounters reactive oxygen species (ROS) in the lungs of CF patients due to the exaggerated, sustained and extended inflammatory response, characterized by influx of neutrophils and high concentrations of interleukin-8 [22–24]. In addition, *P. aeruginosa*-infected CF patients are routinely treated with several types of antibiotics, because early and aggressive antipseudomonal treatment regimens are correlated with improved lung function and survival of CF patients [25]. Recently, two independent studies identified antisense transcription in *P. aeruginosa*. One of the studies investigated strains PAO1 and PA14 at early stationary growth phase and found 60 asRNAs, of which 19 were expressed in strain PAO1 [26]. The other study identified 384 antisense transcriptional start sites, but not full length asRNAs, in *P. aeruginosa* PA14 grown at 28°C and at 37°C [27]. The expression of asRNAs most likely occurs in a transient manner and is dependent on specific environmental cues. Therefore, we chose to monitor the expression of asRNAs in *P. aeruginosa* PAO1 grown in several different conditions to ensure a comprehensive characterization of the *P. aeruginosa* asRNA-ome. Accordingly, exponentially growing populations of *P. aeruginosa* PAO1 were exposed to osmotic, oxidative and antibiotic stress. The antibiotics used in this study were ß-lactams, aminoglycosides, macrolides, colistin and tetracycline. All the antibiotics used, with the exception of tetracyclin, are routinely used against *P. aeruginosa* infections [28–32]. Finally, the expression of asRNAs was also investigated during exponential and stationary growth.

Recently, we showed that library preparation for RNA-Seq plays a fundamental role when aiming at identifying novel transcripts [33]. Using three different RNA-Seq library protocols with different sRNA abundance, we identified over 500 novel intergenic sRNAs in *P. aeruginosa* PAO1 [33, 34]. Although the use of three different libraries increased the number of novel transcripts identified, there were significant differences in the subset of transcripts detected in each library. Libraries that were prepared with a size-selected fraction of RNA were more sensitive in the detection of intergenic sRNAs [33]. In the present study we report 232 novel asRNAs that were identified using strand-specific RNA-Seq libraries that contain cDNA originating from transcripts shorter than 500 nt [33, 34].

## Results and discussion

### Antisense RNAs detection and classification

Exponentially growing cells of *P. aeruginosa* PAO1 were exposed to sodium chloride (NaCl) and hydrogen peroxide (H_2_O_2_) inducing osmotic and oxidative stress conditions, respectively. The antibiotics used were piperacillin, ceftazidime, aztreonam, meropenem, tobramycin, azithromycin, colistin and tetracycline. The concentrations of antibiotics, NaCl and H_2_O_2_ are shown in Table 1. RNA-Seq libraries were prepared using the previously described protocol LIB < 500, which produces strand-specific libraries that contain cDNA originating from transcripts shorter than 500 nt [33, 34]. On average 2.6% of the mapped reads covered regions antisense to previously annotated transcripts (Additional file 1).

**Table 1:** Concentration of antibiotics, NaCl and H_2_O_2_ used in the stress exposure experiments

Condition	Abbreviation	MIC value (μg/ml)	(μg/ml) added at OD = 0.5
Control	**-**	**-**	**-**
Aztreonam	AZT	4	3xMIC
Ceftazidime	CEF	2	3xMIC
Ciprofloxacin	CIP	0.125	3xMIC
Meropenem	MER	1	3xMIC
Tetracyclin	TET	8	3xMIC
Tobramycin	TOB	1	3xMIC
Azithromycin	AZI	1.5	10xMIC
Colistin	CO	4	3xMIC
Piperacillin	PP	4	3xMIC
Hydrogen peroxyde	H_2_O_2_	**-**	1 mM
Sodium chloride	NaCl	**-**	0.5 M

In total, we identified 232 novel asRNAs longer than 50 nt, of which 212 are novel transcripts. The 22 asRNAs already identified by other studies are listed in Tables 2 and 3. Additional file 2 lists the coordinates of the 232 asRNAs, as well as the annotation of genes transcribed from the opposite strand. Only asRNAs longer than 50 nt were taken into consideration. The longest detected asRNA is 581-nt long. The asRNAs were categorized depending on their position with respect to the antisense gene as “start-span” (the asRNA overlaps with the 5’ end of the antisense gene), “internal” (the asRNA starts and ends within the antisense gene), or “end-span” (the asRNA overlaps with 3’ the end of the gene). Thirteen asRNAs overlapped with two contiguous genes transcribed from the opposite strand. Some genes have untranslated regions (UTRs) either in the 5’ or the 3’ end which can contain regulatory elements for controlling gene expression. UTRs sometimes extend into the neighboring genes, so we cannot rule out that some of the detected asRNAs might actually be UTRs from neighboring genes. To prevent mis-annotation of UTRs as asRNAs, we have not included asRNAs that terminated less than 100 bp to the start of a flanking gene with the same direction as the asRNA. However we cannot rule out that some of the novel asRNAs identified in this study might be long UTRs belonging to flanking genes, or even novel coding genes. It is also possible that some of the asRNAs identified are the result of non-specific transcription and thus have no physiological functions.

**Table 2:** **AsRNAs detected in this study and in Ferrara** ***et al.*** **(2012) in** ***P. aeruginosa*** **PAO1** [26]

AsRNA antisense to			This study				Ferrara *et al*. (2012)			
			*P. aeruginosa*PAO1				*P. aeruginosa*PAO1			
Gene name	Gene annotation	Gene direction	Name	Start	End	Length	Name	Start	End	Length
			asRNA	asRNA	asRNA	asRNA	asRNA	asRNA	asRNA	asRNA
*triC*	RND triclosan efflux transporter	>	As6	182516	182617	102	SPA0111	182500	182700	201
***gshB***	**Glutathione synthetase**	**<**	**As19**	**448929**	**449284**	**356**	**SPA0113**	**449000**	**449400**	**401**
PA0667	Conserved hypothetical protein	<	As30	718785	718934	150	SPA0055	719900	720200	301
PA1933	Probable hydroxylase large subunit	>	As96	2113618	2113734	117	SPA0059	2113600	2114100	501
			As97	2113817	2113927	111				
***nuoA***	**NADH dehydrogenase I chain A**	**>**	**As120**	**2982707**	**2982848**	**142**	**SPA0114**	**2982700**	**2982900**	**201**
PA2759	Hypothetical protein	<	As128	3119347	3119453	107	SPA0115	3119200	3119700	501
PA3459	Probable glutamine amidotransferase	>	As152	3866017	3866206	190	SPA0064	3865900	3866200	301
***ponA***	**Penicillin-binding protein 1A**	**>**	**AsponA**	**5680819**	**5681167**	**349**	**SPA0119**	**5680700**	**5681300**	**601**
PA5480	Hypothetical protein	>	As246	6171684	6171915	232	SPA0121	6171700	6172000	301
***ysxC***	**Conserved hypothetical protein**	**>**	**As247**	**6183216**	**6183389**	**174**	**SPA0122**	**6183500**	**6183700**	**201**

The 4 asRNAs detected in all 3 studies are shown in bold.

**Table 3:** **AsRNAs detected in this study and in Wurtzel** ***et al.*** **(2012)** [27]

AsRNA antisense to		This study						Wurtzel *et al.*(2012)		
		*P. aeruginosa*PAO1						*P. aeruginosa*PA14		
Gene name	Gene function	Gene	Gene	Name	Start	End	Length	Gene locus	Gene direction	TSS of asRNA
		locus	direction	asRNA	asRNA	asRNA	asRNA			
*dnaA*	Chromosomal replication initiator protein DnaA	PA0001	>	AsdnaA	1320	1784	465	PA14_00010	>	715
**-**	Hypothetical protein	PA0259	<	As11	290529	290665	137	PA14_03190	<	281975
-	Hypothetical protein	PA0264	<	As13	299094	299181	88	PA14_03420	<	313235
***gshB***	**Glutathione synthetase**	**PA0407**	**<**	**As19**	**448929**	**449284**	**356**	**PA14_05310**	**<**	**463561**
*cupA1*	Fimbrial subunit CupA1	PA2128	>	As102	2342397	2342560	164	PA14_37060	<	3300832
*trxB1*	Thioredoxin reductase 1	PA2616	>	As119	2959629	2959767	139	PA14_30280	<	2622144
***nuoA***	**NADH dehydrogenase I chain A**	**PA2637**	**>**	**As120**	**2982707**	**2982848**	**142**	**PA14_30020**	**<**	**2599053**
*rmf*	Ribosome modulation factor	PA3049	>	As135	3414435	3414491	57	PA14_24650	<	2154716
*fadD2*	Long-chain-fatty-acid--CoA ligase	PA3300	<	As150	3697506	3697616	111	PA14_21340	>	1849717
*fpvB*	Second ferric pyoverdine receptor FpvB	PA4168	>	As174	4666087	4666202	116	PA14_09970	<	856527
*pilY1*	Type 4 fimbrial biogenesis protein PilY1	PA4554	>	As197	5101107	5101200	94	PA14_60310	>	5374009
				As198	5101511	5101611	101			
***ponA***	**Penicillin-binding protein 1A**	**PA5045**	**>**	**AsponA**	**5680819**	**5681167**	**349**	**PA14_66670**	**>**	**5952559**
*wzm*	Membrane subunit of A-band LPS efflux transporter	PA5451	<	As243	6141133	6141239	107	PA14_71960	<	6414559
***ysxC***	**Ribosome biogenesis GTP-binding protein YsxC**	**PA5492**	**>**	**As247**	**6183216**	**6183389**	**174**	**PA14_72480**	**>**	**6456518**

The 4 asRNAs detected in all 3 studies are shown in bold.

**Figure 1:**
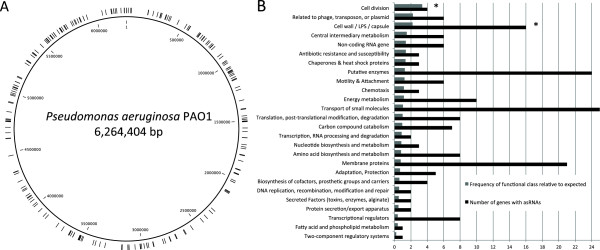
**Distribution and targets of antisense sRNAs. A**. Distribution of antisense sRNAs in the genome of *P. aeruginosa* PAO1. Each asRNA is marked by a black line. **B**. Classification of genes showing antisense transcription according to PseudoCap functional classes. Asterisks (*) denote functional classes that are significantly overrepresented (*P* < 0.05) among the 232 asRNAs.

Next, we examined the distribution of antisense sRNAs in the genome and found the asRNAs to be homogenously distributed throughout the genome of *P. aeruginosa* PAO1 (Figure 1A). A recent study by Wurtzel *et al.* found that antisense sRNAs are more often found in some parts of the accessory genome of *P. aeruginosa* PA14 [27]. Specifically, the authors identified 384 sites with overlapping transcription on the reverse strand, and found that the pathogenicity island PAPI-1 harbors 5-fold more asRNA loci compared to the core genome. In this study we do not observe a similar enrichment of antisense transcripts in certain parts of the genome. There are two reasons that might explain the difference between this study and the one from Wurtzel *et al.* (2012). i) The strain used in the study is *P. aeruginosa* PAO1, while Wurtzel *et al.* (2012) used strain PA14. The PAPI-1 island is found in strain PA14 but not in PAO1, though it can be transferred by a type IV pilus [35]. ii) Wurtzel *et al.* (2012) studied two conditions (growth at 28°C and at 37°C, respectively). In this study we sequenced samples from *P. aeruginosa* growing in 12 different conditions. Studying more conditions might lead to finding antisense transcription sites in more genomic locations, and therefore avoiding enrichment of expression of asRNAs associated to a specific condition.

### Functional classification and differential expression of asRNAs

Figure 1B shows the classification of genes with asRNAs transcribed from the reverse strand in our experiments according to their function. We found a significant overrepresentation of the classes ‘Cell division’ (3.4-fold increase, *P* = 0.03) and ‘Cell wall / LPS / capsule’ (2.2-fold increase, *P* = 0.003). This overrepresentation of genes involved in cell division and cell wall, lipopolysaccharide (LPS), and capsule biosynthesis most likely reflects the conditions used in this study. The conditions studied (stationary phase, osmotic, oxidative and antibiotic stresses) greatly affect both the bacterial replication and cell wall synthesis, and therefore fast regulation of the genes involved in these processes is essential. Indeed we observe significant changes in expression of cell division and LPS genes after the antibiotic treatments (data not shown), which is in agreement with previous studies [36]. Interestingly we have detected 6 asRNAs (as138-as143) that are transcribed opposite to 7 genes of the *wbp* cluster (*wbpBCDE, wzy, wbpG* and *wbpJ*) (Additional file 3). The *wbp* cluster contains genes encoding proteins involved the B-band LPS O-antigen biosynthesis in *P. aeruginosa*, including genes for enzymes involved in synthesis and transfer of sugar nucleotides and O-antigen processing [37]. WbpB, WbpE, and WbpD are the three central enzymes in this pathway [38]. Cirz *et al.* (2006) reported that the transcription of *wbp* genes were decreased by 2- to 6-fold after exposure to ciprofloxacin [36]. Our sequencing results originating from libraries containing the full transcriptome show the same trend after ciprofloxacin and tobramycin exposure, with down-regulation of the *wbp* genes of 2- to 15-fold, and down-regulation of the asRNAs as138-as143 of 2- to 10-fold. However further investigations are required to assess whether these asRNAs affect the levels of the wbp genes encoded on the opposite strand.

**Figure 2:**
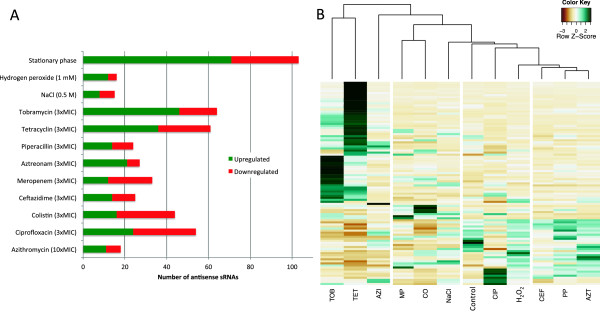
**Differential expression of asRNAs. A**. Number of asRNAs whose expression is significantly changed during the conditions tested. **B**. Heatmap and hierarchical cluster analysis of expression of the most differentially expressed asRNAs in the conditions representing osmotic, oxidative and antibiotic stress compared to the untreated control (*P* < 0.01). Green color represents asRNAs with high expression, and brown color indicates asRNAs with low expression.

Figure 2A represents the number of asRNAs whose expression is significantly changed during the conditions tested (*P* < 0.01). A substantial number of asRNAs significantly change their expression, indicating that their putative regulatory effects may be important during the conditions tested. A hierarchical cluster analysis of expression of the most differentially expressed asRNAs illustrates how related conditions show similar patterns of asRNA expression (Figure 2B). The treatments with three of the β-lactams (CEF, PP, AZT), whose modes of action are alike, are clustered together. The β-lactam not clustered in this group is meropenem, a carbapenem which displays a much faster time-kill curve than the other three β-lactams tested (data not shown). Additional file 4 lists the asRNAs differentially transcribed in our conditions, as well as their fold-change in expression. We anticipate that these data will be important to understand the regulation of genes that show antisense transcription on the reverse strand. In most cases we do not observe a clear and significant correlation between the transcriptional levels of asRNAs and their target genes. This makes it difficult to infer the mode of action of asRNAs, and it might indicate that the role of most asRNAs is to fine-tune the regulation of gene expression. This is known to be the case for some sRNAs in Pseudomonas species [39, 40]. In addition asRNAs can regulate the translation of their target genes either positively or negatively, without affecting their transcriptional levels. Thus the level of expression alone cannot explain the mechanism of action of asRNAs, and more experiments will be needed in order to determine how asRNAs regulate their target genes.

### Comparison between asRNAs detected in different studies

**Figure 3:**
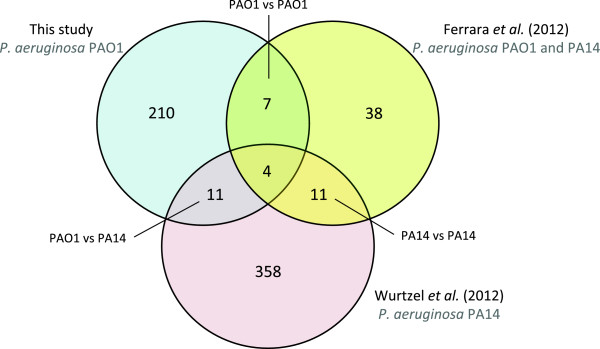
**Comparison between asRNAs detected in different studies.** Venn diagram representing the asRNAs detected in this study, Wurtzel *et al.* (2012) [27] and Ferrara *et al.* (2012) [26].

Recently two independent studies used RNA-Seq to investigate transcription in *P. aeruginosa* and revealed novel asRNAs. One of the studies investigated strains PAO1 and PA14 grown aerobically in Brain Heart Infusion (BHI) rich medium at 37°C and harvested at early stationary phase [26]. The authors found 60 asRNAs. Interestingly, a number of these asRNAs were strain-specific or showed strain-specific expression: 19 asRNAs were only expressed in strain PAO1, 29 were only expressed in strain PA14, and 12 were expressed in both strains. The other study investigated *P. aeruginosa* PA14 grown aerobically at 28°C and at 37°C in LB medium and harvested at early stationary phase [27]. The authors identified 384 transcription start sites with overlapping transcription on the reverse strand. However, due to their library preparation protocol, Wurtzel *et al.* (2012) could not determine the length of the asRNAs, but only the antisense transcription start sites [27]. In this study, we identified 232 asRNAs with lengths ranging from 50 to 581 nt. We did not take into consideration neither asRNAs shorter than 50 nt, nor asRNAs that might be suspected to be UTRs of flanking genes. This might explain why we identify considerably fewer asRNAs than Wurtzel *et al.* (2012). Figure 3 represents the overlap between the asRNAs detected in this study and the two previous ones. Only a small partial overlap between studies is observed. The overlap between pairs of studies ranged from 7 to 11 asRNAs, and only four asRNAs were detected in all three studies (Tables 2, 3 and 4). These asRNAs are transcribed antisense to the glutathione synthetase gene *gshB*, the NADH dehydrogenase I chain A gene *nuoA*, the ribosome biogenesis GTP-binding protein YsxC gene *ysxC,* and the penicillin-binding protein 1A gene *ponA*. We have validated the expression of the latter asRNA, from now on called AsponA, using 5’- and 3’-RACE. The coordinates of *asponA* were very similar between the RNA-Seq data and the RACE experiments, with differences of up to 19 nt between the two techniques. AsponA is upregulated upon aztreonam (2.9-fold), piperacillin (2.8-fold), and ciprofloxacin (2-fold) exposure, and downregulated during meropenem (-2-fold), colistin (-3-fold), tobramycin (-5.4-fold), and tetracycline (-5.6-fold) exposure. AsponA overlaps with the predicted -10 and -35 σ^70^ promoter elements of the *ponA* gene (Additional file 5). Due to the location of AsponA, we hypothesized that it probably belongs to the class of asRNAs that prevent the transcription or translation of the opposite gene, either by transcription interference or by directly blocking ribosome binding. Wurtzel *et al.* (2012) and Ferrara *et al*. (2012) detected the expression of AsponA in *P. aeruginosa* PA14 and PAO1 [26, 27].

**Table 4:** AsRNAs detected in Ferrara *et al.* (2012) [26] and in Wurtzel *et al.* (2012) in *P. aeruginosa* **PA14** [27]

AsRNA antisense to			Ferrara ***et al.***(2012)				Wurtzel ***et al.***(2012)
			***P. aeruginosa***PA14				***P. aeruginosa***PA14
Gene name	Gene function	Gene direction	Name	Start	End	Length	TSS of asRNA
			asRNA	asRNA	asRNA	asRNA	
PA14_04820	TetR family transcriptional regulator	>	SPA0112	425649	426248	600	426072
***gshB***	**Glutathione synthetase**	**<**	**SPA0113**	**463700**	**464100**	**401**	**463561**
PA14_22270	Recombinase	<	SPA0018	1940100	1940300	201	1941053
PA14_28290	Hypothetical protein	>	SPA0116	2446100	2446200	101	2446191
***nuoA***	**NADH dehydrogenase subunit A**	**<**	**SPA0114**	**2599000**	**2599200**	**201**	**2599053**
PA14_35720	Hypothetical protein	<	SPA0019	3176100	3176300	201	3176580
PA14_46460	Hypothetical protein	<	SPA0020	4134300	4134700	401	4134289
*purC*	Phosphoribosylaminoimidazole-succinocarboxamide synthase	<	SPA0168	4553300	4553400	101	4553302
PA14_51540	Transposase	<	SPA0169	4583200	4583500	301	4583062
PA14_59370	Hypothetical protein	<	SPA0021	5288100	5288500	401	5288463
PA14_59580	Transposase	<	SPA0022	5303200	5303500	301	5302248
PA14_59840	Hypothetical protein	>	SPA0023	5330700	5330900	201	5330858
***ponA***	**Penicillin-binding protein 1A**	**>**	**SPA0119**	**5951200**	**5952700**	**1501**	**5952559**
PA14_69050	Hypothetical protein	>	SPA0173	6157800	6158100	301	6157722
***ysxC***	**Ribosome biogenesis GTP-binding protein YsxC**	**>**	**SPA0122**	**6456400**	**6456600**	**201**	**6456518**

The 4 asRNAs detected in all 3 studies are shown in bold.

The lack of overlap between the reported asRNAs may be caused by the different characteristics of each work, as (i) different growth conditions, (ii) different *P. aeruginosa* strains, and (iii) different methods to perform RNA-seq were used in each of them. (i) The three studies investigated different *P. aeruginosa* strains (PAO1 and PA14) grown under different conditions, which may account for part of the differences in the asRNAs detected. As previously mentioned, the level of expression of asRNAs varies greatly that in the different conditions used in our experiments (Figure 2), and in consequence the asRNAs that are detected by RNA-Seq in each condition. (ii) The evolution of bacterial sRNAs appears to be rapid and, as a result, sequence similarities between sRNAs are limited, even between relatively closely related species [41]. In a previous study we showed that, out of 513 novel intergenic sRNAs detected in *P. aeruginosa* PAO1, the sequences of 88% of the sRNAs are not conserved outside *P. aeruginosa,* indicating that the extent of conservation in other Pseudomonas species is limited [33]. Ferrara *et al.* (2012) have reported that even under the same growth conditions, different strains of *P. aeruginosa* (PAO1 and PA14) express different sets of both intergenic and antisense sRNAs [26]. However when comparing studies that investigated the same strain, the extent of the overlap is still small (Figure 3). We considered whether the expression of strain-specific asRNAs was due to the existence of antisense promoters that were present in only one of the two strains. However, upon examination of strain-specific asRNAs, we observed that the predicted antisense promoters were present in both strains even if antisense transcription was only detected in one of them. (iii) We hypothesize that the main cause for the limited overlap between the studies may be the biases arising from the technical variation between the studies. These three studies used considerably different RNA extraction methods, RNA-Seq library preparation protocols, sequencing platforms and bioinformatic analysis pipelines.

Library preparation for RNA-seq experiments requires multiple enzyme-catalyzed steps such as sequential oligonucleotide adapter ligations to the 5’and 3 ends of RNAs, reverse transcription (RT), and PCR. RNA ligase preferences may contribute to the observed bias in sRNA detection [42–45], as well as the RT reaction and PCR [46–48]. The biases in sRNA detection could even be caused by the use of different buffer compositions and additives that modify enzymatic activity. Hafner *et al.* (2011) investigated the biases associated to the adapter ligation steps involved RNA-seq library preparation [42]. The biases found were mainly due to the sequences of RNAs, and that secondary and tertiary structures of RNAs affected the efficiency of 5’- and 3’-adapter ligation during library preparation. The sequences of 5’ and 3’ adapters were also found to affect the ligation yields [42]. In line with these results, another study also demonstrated that RNA and RNA-adapter secondary structures drastically influences RNA ligation efficiencies [43]. Another investigation reported that increased variability of adapter sequences helps to increase the diversity of RNAs ligated [44]. Recent systematic investigations have revealed method-dependent biases in miRNA quantification [49, 50]. Based on identical starting material, these studies demonstrated that alternative library preparation methods resulted in entirely different miRNA expression levels. It was observed that the detection of miRNAs by RNA-seq was strongly biased towards certain miRNAs, preventing the accurate determination of absolute numbers of transcripts [49]. However the biases were found to be systematic and highly reproducible and differential expression results between samples obtained by qPCR and RNA-seq were strongly correlated, showing that RNA-seq is suited for determining relative expression differences between samples [49]. Unexpectedly, the observed bias was mostly independent of the sequencing platform but strongly determined by the method used for library preparation. Library replicates gave similar results, which indicates that the data bias was likely caused by differences inherent to the cDNA preparation protocols [49]. In a previous study, we also generated different RNA-seq libraries to identify bacterial sRNAs [33]. Two libraries only included the RNA fractions shorter than 500 nt and shorter than 200 nt, respectively. Another library contained information on the full transcriptome. Almost all previously known sRNAs and over 500 novel intergenic sRNAs were identified in *P. aeruginosa* with this approach. There were significant differences in the subset of transcripts detected in each library [33], and the libraries that did not include larger transcripts were clearly superior in detecting sRNAs. All these investigations underscore the importance of library preparation strategy and relative sRNA abundance for successful sRNA detection, and show that in order to increase the number of sRNAs detected it is required to apply different parallel sequencing strategies.

## Conclusions

RNA-Seq was used to identify and quantify 232 asRNAs in the opportunistic pathogen *P. aeruginosa* growing under in 13 different conditions, including osmotic, oxidative and antibiotic stress, and exponential and early stationary phase. Due to our library preparation protocol, only asRNAs ranging approximately from 50 to 500 nt were detected. Thus the number of asRNAs provided in this study is most likely an underestimate as longer asRNAs have not been included. These data are important for the understanding of the regulation of genes that show antisense transcription on the reverse strand. However the large number of asRNAs detected makes it difficult to unravel functionality and physiological impacts of antisense transcription, and this paper should therefore be read as a documentation of the potential asRNA repertoire from which specific studies can be designed. In addition, we present data showing that in several cases growth conditions impact on the expression levels of asRNA, which suggest that at least some of the asRNAs may play roles in physiological adaptation to changing conditions. A significant number of asRNAs were transcribed opposite to genes involved in cell division and in cell wall, lipopolysaccharide (LPS), and capsule biosynthesis. A substantial number of antisense sRNAs significantly changed their expression during early stationary phase and under osmotic, oxidative and antibiotic stress, suggesting that asRNAs may play a regulatory role during these conditions.

Finally, we compare our results with those obtained by others in order to document that detection of these new types of potential regulatory molecules is not trivial and that choice of detection and documentation methods is truly important. One of the challenges of performing genome-wide expression studies of sRNAs is to compare the extremely large data sets resulting from different RNA-Seq studies, as library preparation protocols, sequencing platforms, and thresholds for detecting transcripts differ from study to study. Our comparison of three independent studies that used RNA-seq to detect novel asRNAs in *P. aeruginosa* shows that the extent of overlap between the results reported is very limited. It is necessary to address considerations like what is an appropriate threshold of reads for transcript detection, or how reproducibility should be reported in order to avoid misleading conclusions when comparing data generated by non-identical methods. In addition, public databases are already and will be needed for sharing, analyzing, and storing transcriptomic data. Apart from sharing and comparing RNA-seq data, the validation and functional characterization of the hundreds of novel sRNAs now being reported will also be a major challenge of current RNA research.

## Methods

### Growth conditions

Growth in Luria–Bertani (LB) broth (250 rpm, 37°C) or on LB plates at 37°C was used throughout this study. Overnight cultures of *Pseudomonas aeruginosa* PAO1 were diluted to a starting OD_600_ of 0.01 and grown to an OD_600_ of 0.5, at which an antibiotic, 0.5 M NaCl or 1 mM hydrogen peroxide was added. The final concentrations of antibiotics are shown in Table 1. Concentrated stock solutions of H_2_O_2_ and all antibiotics were prepared fresh daily. Cells were harvested 1 hour after the addition of antibiotics, NaCl and H_2_O_2_. Early-stationary phase cells were harvested from cultures grown to an OD_600_ of 3. RNA was extracted and used to make RNA-Seq libraries LIB < 500 (described below). Experiments were performed in duplicates.

### MIC value calculation

Minimum inhibitory concentrations (MIC) of *P. aeruginosa* PAO1 were assessed using both the broth microdilution procedure and E-test strips.

*Broth microdilution.* LB medium was added to all wells of a 96-well microtiter plate loaded with serially diluted antibiotics. Each well was inoculated with *P. aeruginosa* PAO1 at a final concentration of 5 × 10^5^ cfu/ml. The plates were incubated for 24 hours (250 rpm, 37°C). Following incubation, the optical density of all wells was measured and the lowest concentration showing complete inhibition of growth was recorded as the MIC for that antibiotic. The experiments were performed in triplicates. Concentrated stock solutions of all antibiotics were prepared fresh daily.

*E-Test.* E-test (bioMérieux AB BIODISK) strips were used according to the manufacturer’s instructions. LB plates were inoculated equal amount of *P. aeruginosa* PAO1 cells. After drying, the E-test strips were placed on the top of the plates. The MIC values were read after 16 h of incubation at 37°C. The experiments were performed in triplicates.

### RNA isolation

Harvested cells were mixed immediately with 0.2 volumes of STOP solution (95% ethanol, 5% phenol) and pelleted by centrifugation. Total RNA was extracted with Trizol (Invitrogen). Removal of DNA was carried out by treatment with DNase I (Fermentas) in combination with the RNase inhibitor RiboLock (Fermentas). The integrity of total RNA and DNA contamination were assessed with an Agilent 2100 Bioanalyzer (Agilent Technologies).

### Removal of 23S, 16S and 5S rRNAs

The 23S, 16S and 5S rRNAs were removed by subtractive hybridization using the MICROBExpress kit (Ambion) with modifications as previously described [33, 34]. Capture oligonucleotides complementary to the rRNAs of *P. aeruginosa* PAO1 were used (Additional file 6). Removal of rRNAs was confirmed with an Agilent 2100 Bioanalyzer (Agilent Technologies).

### Library preparation and RNA sequencing

Sequencing libraries were constructed as previously described, following LIB < 500 and LIB > 100 protocols [33, 34]. Each type of library was prepared in duplicate using RNA extracted from biological replicates. Libraries LIB < 500 are strand-specific and contain cDNAs originating from transcripts shorter than 500 nt. Libraries LIB > 500 were used for asRNA detection. RNA size selection was performed by running total RNA on 10% polyacrylamide gels containing 10 M urea. Gel slices corresponding to RNAs up to 500 nt were excised, followed by elution of RNA in 0.4 M NaCl and precipitation with ethanol. The 5S rRNA was depleted as previously described, followed by treatment with Tobacco Acid Pyrophosphatase (Epicentre Technologies) at 37°C for 90 min. Tobacco Acid Pyrophosphatase (TAP) is used to convert 5’-triphosphate RNA into 5’-monophosphate RNA, which is important for correct adapter ligation. This was followed by treatment with RNase III (Ambion) for 10 min at 37°C to fragment the RNA. RNase III fragments RNA into smaller pieces containing a 5’-phosphoryl group and a 3’-hydroxyl group, which is important for specific adapter ligation in the next step. Sequential ligation of RNA 3’ and 5’ adapters was performed using the adapters and enzymes from the TruSeq Small RNA Sample Preparation kit (Illumina). Next, reverse transcription followed by PCR amplification was performed to form cDNA constructs based on the RNA fragments ligated with 3’ and 5’ adapters, selectively enriching fragments with adapter molecules on both ends. The reverse transcription and subsequent PCR amplification were performed using the enzymes and reagents from the TruSeq Small RNA Sample Preparation kit (Illumina). Libraries LIB > 100 contain cDNA from all RNAs transcribed with the exception of 23S, 16S and 5S rRNAs. This type of library was prepared using using the TruSeq RNA Sample Preparation kit (Illumina) following the manufacturer’s instructions. Agencourt AMPure XP beads (Beckman Coulter Genomics) were used for the post-PCR clean-up. After each step the samples were validated using an Agilent 2100 Bioanalyzer (Agilent Technologies), and the final concentration was measured using a Qubit 2.0 Fluorometer (Invitrogen). The libraries were sequenced using the Illumina HiSeq2000 platform with a paired-end protocol and read lengths of 100 nt.

### Data analysis

Our analysis pipeline is described in detail in Gómez-Lozano *et al.* (2014) [34]. Briefly, reads were mapped onto the *P. aeruginosa* PAO1 genome (RefSeq Accession No. NC_002516) using the Bowtie 2 short read aligner [51]. Read alignments from Bowtie 2 were handled using SAMtools [52]. Information about the number of reads of each library used for asRNA detection can be found in Additional file 1. In order to obtain normalized expression intensities of the read coverage depth at each position in the genome, the number of reads in each replicate was normalized according to the total number of reads in the library, and expression intensities from replicate samples were averaged. A custom-made script was designed to detect novel transcripts antisense to annotated genes (Additional file 7) [34]. Only transcripts of at least 50 nt were considered further. The resulting transcripts from automatic classification were re-evaluated by visual inspection. An analysis of variance (ANOVA) was performed on the average expression of the transcripts to determine those with differential expression between the conditions tested (*P*-value <0.05 and fold change 2). Sequencing libraries LIB < 500 have been submitted to the European Nucleotide Archive under accession number PRJEB6998 (http://www.ebi.ac.uk/ena/data/view/PRJEB6998).

### Rapid amplification of cDNA ends (RACE)

Additional file 6 lists primers and adapters used.

*5’ RACE.* 2 μg rRNA-depleted RNA was incubated with 10 U of Tobacco Acid Pyrophosphatase (Epicentre Technologies) at 37°C for 1 h to convert RNA 5’ triphosphates in 5’ monophosphates. The same amount of control RNA was incubated under the same conditions in the absence of the enzyme. Reactions were stopped by phenol-chloroform extraction, followed by etanol-sodium acetate precipitation. Precipitated RNAs were redissolved in water, mixed with 500 pmol of 5’ RNA adapter, heat-denatured at 95°C for 5 min, then quick-chilled on ice. A short RNA adapter was ligated was ligated with 50 U of T4 RNA ligase (Thermo Scientific) at 37°C for 1 h. Reactions were stopped by phenol-chloroform extraction, followed by ethanol–sodium acetate precipitation. Precipitated RNAs were re-dissolved in 20 μl water. Then 10 μl of 5’adapter-ligated RNA was reverse-transcribed using 2 pmol of primer complementary to the sRNA (5’-GSP1) and the Thermoscript RT-PCR system (Invitrogen) according to the manufacturer’s instructions. Reverse transcription (RT) was performed in three subsequent 20 min steps at 55°C, 60°C, and 65°C, followed by treatment with RNase H. Primers were removed using the NucleoSpin Gel and PCR Clean-up kit (Macherey-Nagel). The products of RT were amplified using 10 pmol of another primer complementary to the sRNA (5’-GSP2) and 10 pmol the 5’ adapter-specific primer, together with the Maxima Hot Start PCR Master Mix (Thermo Scientific) according to the manufacturer’s instructions. Negative controls were performed using the 5’ adapter-ligated RNA as template. The PCR products were resolved and purified using E-Gel SizeSelect 2% Agarose gels (Invitrogen). Products were sequenced with 5’-GSP2 and 5’ adapter-specific primers by LGC Genomics GmbH (Germany).

*3’ RACE*. 2 μg rRNA-depleted RNA was dephosphorylated with calf intestinal alkaline phosphatase (New England Biolabs) at 37°C for 1 h. Reactions were stopped by phenol-chloroform extraction, followed by etanol-sodium acetate precipitation. Ligation was done as above with a 3’ RNA adapter with a 3’-inverted deoxythymidine (3’-idT). RT was carried out as described, but with 10 pmol of a single primer complementary to the 3’ RNA adapter. PCR amplification, band purification and sequence analysis was done as described above. All enzymatic treatments of RNA were performed in the presence of 20 units of RiboLock RNase Inhibitor (Thermo Scientific).

## Electronic supplementary material

Additional file 1: **Information about sequencing libraries.** (PDF 71 KB)

Additional file 2: **Annotation of antisense sRNAs.** (PDF 107 KB)

Additional file 3: **Antisense transcription in the** ***wbp*** **operon.** (PDF 74 KB)

Additional file 4: **Antisense sRNAs differentially expressed.** (PDF 83 KB)

Additional file 5: **AsponA in** ***P. aeruginosa*** **PAO1.** (PDF 104 KB)

Additional file 6: **Primers and adapters used in the study.** (PDF 72 KB)

Additional file 7: **Script for extracting antisense reads.** (PDF 136 KB)
