# Supplementary material for: Expression of antisense small RNAs in response to stress in Pseudomonas aeruginosa
Source: BMC Genomics. 2014 Sep 11;15(1):783. doi: 10.1186/1471-2164-15-783 (PMC4180829; doi:10.1186/1471-2164-15-783)
Supplement: Supplementary file 1 — Additional file 1: Information about sequencing libraries. (PDF 71 KB) [file 12864_2014_6485_MOESM1_ESM.pdf]

Additional file 1. Information about sequencing libraries LIB<500 used for asRNA detection.

Condition	Number of biological replicates	Library Name	Total number of read pairs	Number of mapped read pairs	Number of mapped read pairs to antisense regions
Exponential growth (control)	2	EXP_1	8990460	4504801	155702
		EXP_2	9709285	5147313	150599
Stationary growth	2	STA_1	7323352	5756111	318667
		STA_2	7822811	6022734	343947
Azithromycin	2	AZI_1	9262216	5264644	94551
		AZI_2	10046880	5942905	149304
Aztreonam	2	AZT_1	11022005	5797896	194128
		AZT_2	9732194	5995572	191271
Ceftazidime	2	CEF_1	9810278	4408864	122171
		CEF_2	9474852	5899270	153214
Ciprofloxacin	2	CIP_1	10962090	4725534	127966
		CIP_2	10548496	5318724	154348
Colistin	2	CO_1	8296145	5892397	56656
		CO_2	9578219	6648768	60274
Hydrogen peroxyde	2	H2O2_1	9045487	5337832	179901
		H2O2_2	9698657	5519753	214202
Meropenem	2	MP_1	8992185	6016045	79446
		MP_2	10749222	6998403	78118
Sodium chloride	2	NaCl_1	8554400	4769991	102896
		NaCl_2	10865953	5893518	99559
Piperacillin	2	PP_1	8126685	5068797	167740
		PP_2	8217552	5326984	176163
Tetracyclin	2	TET_1	8466108	5014247	67706
		TET_2	9218538	5083513	68507
Tobramycin	2	TOB_1	8807143	4899450	85418
		TOB_2	8915533	5912559	98559
