# Supplementary material for: Expression of antisense small RNAs in response to stress in Pseudomonas aeruginosa
Source: BMC Genomics. 2014 Sep 11;15(1):783. doi: 10.1186/1471-2164-15-783 (PMC4180829; doi:10.1186/1471-2164-15-783)
Supplement: Supplementary file 2 — Additional file 2: Annotation of antisense sRNAs. (PDF 107 KB) [file 12864_2014_6485_MOESM2_ESM.pdf]

Additional file 2. Annotation of antisense sRNAs.

Name	Start*	End*	Length*	Type of sRNA**	Antisense gene***	Direction gene	Gene name	Gene function	Gene pathway
asdnaA****	1320	1784	465	internal	PA0001	>	*dnaA*	chromosomal replication initiator protein DnaA	DNA replication, recombination, modification and repair
as4	35866	35974	109	end-span	PA0033	<	*hptC*	Histidine phosphotransfer protein HptC	
as5	40775	40925	151	end-span	PA0039	>		hypothetical protein	Hypothetical, unclassified, unknown
as6	182516	182617	102	end-span	PA0158	>	*triC*	Resistance-Nodulation-Cell Division (RND) triclosan efflux transporter, TriC	Antibiotic resistance and susceptibility; Transport of small molecules
as7	184720	184861	142	internal	PA0162	>	*opdC*	histidine porin OpdC	Membrane proteins; Transport of small molecules
as8	226915	226976	62	internal	PA0196	>	*pntB*	pyridine nucleotide transhydrogenase, beta subunit	Transport of small molecules; Energy metabolism
as9	263722	263909	188	internal	PA0234	>		hypothetical protein	Hypothetical, unclassified, unknown
as10	288493	288639	147	internal	PA0257	<		hypothetical protein	Related to phage, transposon, or plasmid
as11	290529	290665	137	internal	PA0259	<		hypothetical protein	Hypothetical, unclassified, unknown
as12	291428	291543	116	internal	PA0260	<		hypothetical protein	Hypothetical, unclassified, unknown; Membrane proteins
as13	299094	299181	88	internal	PA0264	<		hypothetical protein	Hypothetical, unclassified, unknown
as14	334305	334355	51	internal	PA0296	<	*spuI*	Glutamylpolyamine synthetase	Putative enzymes; Carbon compound catabolism
as15	338651	338736	86	internal	PA0300	>	*spuD*	polyamine transport protein	Transport of small molecules
as16	353325	353458	134	internal	PA0313	<		L-cysteine transporter of ABC system YecS	Membrane proteins; Transport of small molecules
as17	368517	368669	153	internal	PA0328	<		hypothetical protein	Hypothetical, unclassified, unknown
as18	379215	379350	136	internal	PA0337	>	*ptsP*	phosphoenolpyruvate-protein phosphotransferase PtsP	Transport of small molecules
as19	448929	449284	356	internal	PA0407	<	*gshB*	glutathione synthetase	Amino acid biosynthesis and metabolism; Biosynthesis of cofactors, prosthetic groups and carriers
as20	521726	521838	113	internal	PA0461	<		conserved hypothetical protein	Hypothetical, unclassified, unknown
as21	544658	544778	121	internal	PA0483	>		probable acetyltransferase	Putative enzymes
as22	554963	555078	116	internal	PA0495	>		hypothetical protein	Hypothetical, unclassified, unknown
as23	557312	557569	258	end-span	PA0498	<		hypothetical protein	Hypothetical, unclassified, unknown
as24	646779	646892	114	internal	PA0588	<		conserved hypothetical protein	Hypothetical, unclassified, unknown
as25	654775	654912	138	internal	PA0595	<	*ostA*	organic solvent tolerance protein OstA precursor	Adaptation, Protection
as26	659114	659270	157	internal	PA0599	<		hypothetical protein	Hypothetical, unclassified, unknown
as27	680736	680853	118	internal	PA0620	>		probable bacteriophage protein	Related to phage, transposon, or plasmid
as28	697609	697703	95	internal	PA0641	>		probable bacteriophage protein	Related to phage, transposon, or plasmid
as29	701233	701489	257	end-span	PA0645	>		hypothetical protein	Related to phage, transposon, or plasmid
as29	701233	701489	257	start-span	PA0646	>		hypothetical protein	Related to phage, transposon, or plasmid
as30	718785	718934	150	internal	PA0667	<		conserved hypothetical protein	Hypothetical, unclassified, unknown
as31	748895	749047	153	internal	PA0689	>		hypothetical protein	Hypothetical, unclassified, unknown; Transport of small molecules
as32	749460	749562	103	internal	PA0689	>		hypothetical protein	Hypothetical, unclassified, unknown; Transport of small molecules
as33	761761	761823	63	internal	PA0690	>		hypothetical protein	Hypothetical, unclassified, unknown
as34	784213	784303	91	internal	PA0713	>		hypothetical protein	Hypothetical, unclassified, unknown
as35	795503	795648	146	internal	PA0727	>		hypothetical protein from bacteriophage Pf1	Hypothetical, unclassified, unknown; Related to phage, transposon, or plasmid
as36	798624	798854	231	start-span	PA0730	<		probable transferase	Putative enzymes
as37	817964	818095	132	start-span	PA0750	>	*ung*	uracil-DNA glycosylase	DNA replication, recombination, modification and repair
as38	898637	898918	282	end-span	PA0823	<		hypothetical protein	Hypothetical, unclassified, unknown
as38	898637	898918	282	start-span	PA0822	<		hypothetical protein	Hypothetical, unclassified, unknown
as39	921673	921840	168	end-span	PA0845	<		conserved hypothetical protein	Hypothetical, unclassified, unknown
as40	967079	967178	100	internal	PA0884	>		probable C4-dicarboxylate-binding periplasmic protein	Transport of small molecules
as41	967184	967283	100	internal	PA0884	>		probable C4-dicarboxylate-binding periplasmic protein	Transport of small molecules
as42	977508	977658	151	internal	PA0894	>		hypothetical protein	Hypothetical, unclassified, unknown
as43	990194	990309	116	internal	PA0904	>	*lysC*	aspartate kinase alpha and beta chain	Amino acid biosynthesis and metabolism
as44	1026460	1026563	104	internal	PA0936	<	*lpxO2*	lipopolysaccharide biosynthetic protein LpxO2	Putative enzymes; Cell wall / LPS / capsule
as45	1027896	1028083	188	end-span	PA0937	>		conserved hypothetical protein	Hypothetical, unclassified, unknown
as47	1056103	1056248	146	internal	PA0972	>	*tolB*	TolB protein	Transport of small molecules
as48	1060351	1060676	326	end-span	PA0977	<		hypothetical protein	Hypothetical, unclassified, unknown
as49	1066483	1066610	128	internal	PA0985	<	*pyoS5*	pyocin S5	Membrane proteins; Secreted Factors (toxins, enzymes, alginate)
as50	1073865	1074007	143	end-span	PA0992	>	*cupC1*	fimbrial subunit CupC1	Motility & Attachment
as50	1073865	1074007	143	start-span	PA0993	>	*cupC2*	chaperone CupC2	Motility & Attachment; Chaperones & heat shock proteins
as52	1098349	1098557	209	internal	PA1015	<		probable transcriptional regulator	Transcriptional regulators
as53	1119615	1119886	272	end-span	PA1032	<	*quiP*	QuiP	Carbon compound catabolism; Putative enzymes; Antibiotic resistance and susceptibility
as54	1135102	1135286	185	end-span	PA1047	>		probable esterase	Putative enzymes
as55	1135776	1135875	100	internal	PA1048	>		probable outer membrane protein precursor	Membrane proteins; Transport of small molecules
as56	1141250	1141364	115	start-span	PA1053	>		conserved hypothetical protein	Membrane proteins
as57	1207482	1207531	50	internal	PA1113	>		probable ATP-binding/permease fusion ABC transporter	Membrane proteins; Transport of small molecules
as58	1232028	1232169	142	start-span	PA1140	<		conserved hypothetical protein	Hypothetical, unclassified, unknown
as59	1323807	1323918	112	internal	PA1221	<		hypothetical protein	Hypothetical, unclassified, unknown
as60	1394817	1395002	186	internal	PA1284	<		probable acyl-CoA dehydrogenase	Putative enzymes
as62	1455941	1456046	106	internal	PA1342	<		probable binding protein component of ABC transporter	Transport of small molecules
as64	1484863	1485037	175	internal	PA1370	>		hypothetical protein	Hypothetical, unclassified, unknown
as65	1485541	1485595	55	internal	PA1370	>		hypothetical protein	Hypothetical, unclassified, unknown
as66	1499584	1499684	101	internal	PA1382	>		probable type II secretion system protein	Protein secretion/export apparatus
as67	1499690	1499742	53	internal	PA1382	>		probable type II secretion system protein	Protein secretion/export apparatus
as68	1502841	1502987	147	internal	PA1383	>		hypothetical protein	Hypothetical, unclassified, unknown
as69	1503570	1503696	127	internal	PA1384	>	*galE*	UDP-glucose 4-epimerase	Nucleotide biosynthesis and metabolism; Carbon compound catabolism; Central intermediary metabolism
as70	1503843	1503937	95	internal	PA1384	>	*galE*	UDP-glucose 4-epimerase	Nucleotide biosynthesis and metabolism; Carbon compound catabolism; Central intermediary metabolism
as71	1504553	1504676	124	end-span	PA1384	>	*galE*	UDP-glucose 4-epimerase	Nucleotide biosynthesis and metabolism; Carbon compound catabolism; Central intermediary metabolism
as71	1504553	1504676	124	start-span	PA1385	>		probable glycosyl transferase	Cell wall / LPS / capsule
as72	1504788	1504927	140	internal	PA1385	>		probable glycosyl transferase	Cell wall / LPS / capsule
as73	1506107	1506208	102	internal	PA1386	>		probable ATP-binding component of ABC transporter	Transport of small molecules
as74	1558500	1558752	253	internal	PA1430	>	*lasR*	transcriptional regulator LasR	Adaptation, Protection; Transcriptional regulators
as75	1588434	1588549	116	internal	PA1458	>		probable two-component sensor	Chemotaxis; Two-component regulatory systems
as76	1639661	1639805	145	end-span	PA1510	<		hypothetical protein	Hypothetical, unclassified, unknown
as76	1639661	1639805	145	start-span	PA1509	<		hypothetical protein	Hypothetical, unclassified, unknown
as77	1640773	1640948	176	internal	PA1510	<		hypothetical protein	Hypothetical, unclassified, unknown
as78	1698424	1698534	111	internal	PA1561	<	*aer*	aerotaxis receptor Aer	Adaptation, Protection; Chemotaxis
as79	1717815	1717930	116	end-span	PA1578	>		hypothetical protein	Hypothetical, unclassified, unknown
as80	1732127	1732265	139	end-span	PA1589	>	*sucD*	succinyl-CoA synthetase alpha chain	Energy metabolism
as81	1785747	1785881	135	internal	PA1640	<		conserved hypothetical protein	Hypothetical, unclassified, unknown
as82	1790530	1790650	121	internal	PA1645	<		hypothetical protein	Hypothetical, unclassified, unknown
as83	1838134	1838246	113	internal	PA1688	>		hypothetical protein	Hypothetical, unclassified, unknown
as84	1911985	1912118	134	internal	PA1767	<		hypothetical protein	Membrane proteins
as85	1912994	1913099	106	end-span	PA1769	<		conserved hypothetical protein	Hypothetical, unclassified, unknown
as86	1913728	1913919	192	start-span	PA1769	<		conserved hypothetical protein	Hypothetical, unclassified, unknown
as87	1916046	1916172	127	internal	PA1770	>	*ppsA*	phosphoenolpyruvate synthase	Energy metabolism; Carbon compound catabolism; Central intermediary metabolism
as88	1949036	1949142	107	internal	PA1797	<		hypothetical protein	Hypothetical, unclassified, unknown
as89	1953270	1953371	102	internal	PA1800	>	*tig*	trigger factor	Cell division; Chaperones & heat shock proteins
as90	1954335	1954415	81	internal	PA1801	>	*clpP*	ClpP	Chaperones & heat shock proteins; Cell wall / LPS / capsule

as91	1954420	1954469	50	internal	PA1801	>	*clpP*	ClpP	Chaperones & heat shock proteins; Cell wall / LPS / capsule
as92	2012282	2012454	173	internal	PA1852	<		hypothetical protein	Hypothetical, unclassified, unknown
as93	2027111	2027193	83	internal	PA1866	<		hypothetical protein	Hypothetical, unclassified, unknown
as94	2027457	2027571	115	internal	PA1866	<		hypothetical protein	Hypothetical, unclassified, unknown
as95	2113335	2113442	108	internal	PA1933	>		probable hydroxylase large subunit	Carbon compound catabolism
as96	2113618	2113734	117	internal	PA1933	>		probable hydroxylase large subunit	Carbon compound catabolism
as97	2113817	2113927	111	internal	PA1933	>		probable hydroxylase large subunit	Carbon compound catabolism
as98	2120607	2120921	315	internal	PA1939	<		hypothetical protein	Hypothetical, unclassified, unknown
as99	2121071	2121240	170	internal	PA1939	<		hypothetical protein	Hypothetical, unclassified, unknown
as100	2128729	2128826	98	internal	PA1944	>		hypothetical protein	Hypothetical, unclassified, unknown
as101	2314771	2314932	162	internal	PA2103	>		probable molybdopterin biosynthesis protein MoeB	Biosynthesis of cofactors, prosthetic groups and carriers
as102	2342397	2342560	164	start-span	PA2128	>	*cupA1*	fimbrial subunit CupA1	Motility & Attachment
as103	2400697	2400826	130	internal	PA2179	<		hypothetical protein	Hypothetical, unclassified, unknown
as104	2445134	2445251	118	internal	PA2222	<		hypothetical protein	Hypothetical, unclassified, unknown
as105	2550592	2550780	189	start-span	PA2313	>		hypothetical protein	Membrane proteins
as107	2623295	2623503	209	internal	PA2372	>		hypothetical protein	Hypothetical, unclassified, unknown
as108	2701170	2701296	127	start-span	PA2418	>		hypothetical protein	Hypothetical, unclassified, unknown
as110	2756154	2756267	114	end-span	PA2455	>		hypothetical protein	Hypothetical, unclassified, unknown
as111	2756364	2756944	581	end-span	PA2456	>		hypothetical protein	Hypothetical, unclassified, unknown
as111	2756364	2756944	581	start-span	PA2457	>		hypothetical protein	Hypothetical, unclassified, unknown
as113	2802778	2802933	156	internal	PA2484	<		conserved hypothetical protein	Hypothetical, unclassified, unknown
as114	2818927	2819159	233	end-span	PA2502	<		hypothetical protein	Hypothetical, unclassified, unknown
as115	2902292	2902391	100	internal	PA2567	>		hypothetical protein	Hypothetical, unclassified, unknown
as117	2946138	2946205	68	internal	PA2603	>		tRNA-Ser	Putative enzymes
as119	2959629	2959767	139	internal	PA2616	>	*trxB1*	thioredoxin reductase 1	Nucleotide biosynthesis and metabolism
as120	2982707	2982848	142	start-span	PA2637	>	*nuoA*	NADH dehydrogenase I chain A	Energy metabolism
as121	3005381	3005497	117	internal	PA2654	>		probable chemotaxis transducer	Adaptation, Protection; Chemotaxis
as122	3013744	3013890	147	start-span	PA2664	<	*fhp*	flavohemoprotein	Energy metabolism
as123	3015850	3015969	120	end-span	PA2666	>		probable 6-pyruvoyl tetrahydrobiopterin synthase	Biosynthesis of cofactors, prosthetic groups and carriers
as124	3016813	3016931	119	start-span	PA2668	<		hypothetical protein	Hypothetical, unclassified, unknown
as125	3060103	3060257	155	start-span	PA2705	<		hypothetical protein	Hypothetical, unclassified, unknown
as126	3110332	3110396	65	internal	PA2748	<		probable methionine aminopeptidase	Translation, post-translational modification, degradation
as127	3110413	3110473	61	internal	PA2748	<		probable methionine aminopeptidase	Translation, post-translational modification, degradation
as128	3119347	3119453	107	end-span	PA2759	<		hypothetical protein	Hypothetical, unclassified, unknown
as129	3120806	3120915	110	internal	PA2760	>	*oprQ*	OprQ	
as131	3173549	3173835	287	end-span	PA2819	>		tRNA-Glu	Hypothetical, unclassified, unknown
as132	3312901	3313118	218	internal	PA2953	>		electron transfer flavoprotein-ubiquinone oxidoreductase	Energy metabolism
as133	3357890	3357996	107	internal	PA3000	<	*aroP1*	aromatic amino acid transport protein AroP1	Transport of small molecules
as134	3395484	3395684	201	internal	PA3032	<	*snr1*	cytochrome c Snr1	Energy metabolism
as135	3414435	3414491	57	internal	PA3049	>	*rmf*	ribosome modulation factor	Translation, post-translational modification, degradation
as136	3508613	3508673	61	internal	PA3125	>		hypothetical protein	Hypothetical, unclassified, unknown
as137	3517655	3517722	68	internal	PA3135	<		probable transcriptional regulator	Transcriptional regulators
as138	3530607	3530794	188	internal	PA3147	<	*wbpJ*	probable glycosyl transferase WbpJ	Putative enzymes; Cell wall / LPS / capsule
as139	3534247	3534324	78	internal	PA3150	<	*wbpG*	LPS biosynthesis protein WbpG	Cell wall / LPS / capsule
as140	3539090	3539161	72	end-span	PA3155	<	*wbpE*	UDP-2-acetamido-2-dideoxy-d-ribo-hex-3-uluronic acid transaminase, wbpE	Putative enzymes; Cell wall / LPS / capsule
as140	3539090	3539161	72	start-span	PA3154	<	*wzy*	B-band O-antigen polymerase	Membrane proteins; Cell wall / LPS / capsule
as141	3540497	3540593	97	internal	PA3156	<	*wbpD*	UDP-2-acetamido-3-amino-2,3-dideoxy-d-glucuronic acid N-acetyltransferase, WbpD	Putative enzymes; Cell wall / LPS / capsule
as142	3540908	3541027	120	internal	PA3157	<	*wbpC*	probable acetyltransferase	Putative enzymes; Cell wall / LPS / capsule
as143	3542803	3542997	195	internal	PA3158	<	*wbpB*	UDP-2-acetamido-2-deoxy-d-glucuronic acid 3-dehydrogenase, WbpB	Putative enzymes; Cell wall / LPS / capsule
as144	3592018	3592128	111	end-span	PA3200	<		conserved hypothetical protein	Hypothetical, unclassified, unknown
as144	3592018	3592128	111	start-span	PA3199	<		conserved hypothetical protein	Hypothetical, unclassified, unknown
as145	3593054	3593188	135	start-span	PA3201	>		conserved hypothetical protein	Membrane proteins
as146	3612043	3612282	240	internal	PA3224	<		hypothetical protein	Hypothetical, unclassified, unknown
as147	3625886	3625995	110	internal	PA3238	>		hypothetical protein	Hypothetical, unclassified, unknown
as148	3626660	3626776	117	internal	PA3239	>		conserved hypothetical protein	Hypothetical, unclassified, unknown
as149	3631510	3631629	120	end-span	PA3243	>	*minC*	cell division inhibitor MinC	Cell division
as149	3631510	3631629	120	start-span	PA3244	>	*minD*	cell division inhibitor MinD	Cell division
as150	3697506	3697616	111	internal	PA3300	<	*fadD2*	long-chain-fatty-acid--CoA ligase	Fatty acid and phospholipid metabolism
as151	3752069	3752155	87	internal	PA3341	<		probable transcriptional regulator	Transcriptional regulators
as152	3866017	3866206	190	internal	PA3459	>		probable glutamine amidotransferase	Amino acid biosynthesis and metabolism
as154	3944227	3944342	116	internal	PA3524	>	*gloA1*	lactoylglutathione lyase	Central intermediary metabolism
as155	3999082	3999164	83	end-span	PA3567	>		probable oxidoreductase	Putative enzymes
as156	4014211	4014326	116	internal	PA3582	>	*glpK*	glycerol kinase	Central intermediary metabolism
as157	4048252	4048377	126	start-span	PA3613	<		hypothetical protein	Hypothetical, unclassified, unknown
as158	4070600	4070735	136	internal	PA3636	<	*kdsA*	2-dehydro-3-deoxyphosphooctonate aldolase	Carbon compound catabolism; Cell wall / LPS / capsule
as159	4087208	4087269	62	internal	PA3648	<	*opr86*	outer membrane protein Opr86	Membrane proteins; Transport of small molecules
as160	4092478	4092590	113	internal	PA3654	<	*pyrH*	uridylate kinase	Nucleotide biosynthesis and metabolism
as161	4110202	4110321	120	internal	PA3670	<		hypothetical protein	Hypothetical, unclassified, unknown
as162	4156819	4156946	128	internal	PA3711	>		probable transcriptional regulator	Transcriptional regulators
as163	4166790	4167046	257	internal	PA3721	>	*nalC*	NalC	Transcriptional regulators; Antibiotic resistance and susceptibility
as164	4185534	4185601	68	internal	PA3734	>		hypothetical protein	Hypothetical, unclassified, unknown
as165	4242856	4242966	111	internal	PA3783	>		hypothetical protein	Hypothetical, unclassified, unknown
as166	4311907	4312074	168	end-span	PA3851	<		hypothetical protein	Hypothetical, unclassified, unknown
as167	4327946	4328181	236	internal	PA3866	<		pyocin protein	Adaptation, Protection; Secreted Factors (toxins, enzymes, alginate)
as168	4328357	4328518	162	internal	PA3866	<		pyocin protein	Adaptation, Protection; Secreted Factors (toxins, enzymes, alginate)
as169	4329401	4329645	245	internal	PA3866	<		pyocin protein	Adaptation, Protection; Secreted Factors (toxins, enzymes, alginate)
as171	4471922	4472053	132	internal	PA3991	<		hypothetical protein	Hypothetical, unclassified, unknown
as172	4616918	4616968	51	internal	PA4128	>		conserved hypothetical protein	Putative enzymes
as173	4619821	4619903	83	internal	PA4131	>		probable iron-sulfur protein	Putative enzymes
as174	4666087	4666202	116	internal	PA4168	>	*fpvB*	second ferric pyoverdine receptor FpvB	Transport of small molecules
as175	4711769	4711861	93	internal	PA4208	>	*opmD*	probable outer membrane protein precursor	Membrane proteins; Transport of small molecules
as176	4759525	4759686	162	end-span	PA4247	<	*rplR*	50S ribosomal protein L18	Translation, post-translational modification, degradation
as176	4759525	4759686	162	start-span	PA4246	<	*rpsE*	30S ribosomal protein S5	Translation, post-translational modification, degradation
as178	4761521	4761592	72	internal	PA4251	<	*rplE*	50S ribosomal protein L5	Translation, post-translational modification, degradation
as179	4763739	4763790	52	internal	PA4257	<	*rpsC*	30S ribosomal protein S3	Translation, post-translational modification, degradation
as180	4764919	4765009	91	internal	PA4260	<	*rplB*	50S ribosomal protein L2	Translation, post-translational modification, degradation
as181	4773392	4773503	112	internal	PA4269	<	*rpoC*	DNA-directed RNA polymerase beta* chain	Transcription, RNA processing and degradation
as182	4780416	4780494	79	internal	PA4270	<	*rpoB*	P26	Transcription, RNA processing and degradation
as183	4781848	4781947	100	internal	PA4272.1	>	*P27*	P27	Non-coding RNA gene
as184	4842427	4842583	157	end-span	PA4313	>		hypothetical protein	Hypothetical, unclassified, unknown
as185	4919101	4919236	136	internal	PA4389	>		probable short-chain dehydrogenase	Putative enzymes; Amino acid biosynthesis and metabolism
as186	4925623	4925733	111	internal	PA4395	<		conserved hypothetical protein	Hypothetical, unclassified, unknown
as187	4982939	4983086	148	internal	PA4448	<	*hisD*	histidinol dehydrogenase	Amino acid biosynthesis and metabolism
as188	4991277	4991330	54	internal	PA4459	>		conserved hypothetical protein	Hypothetical, unclassified, unknown
as189	5002347	5002404	58	internal	PA4473	>		hypothetical protein	Hypothetical, unclassified, unknown
as190	5018832	5019000	169	internal	PA4488	<		conserved hypothetical protein	Hypothetical, unclassified, unknown
as191	5037299	5037430	132	internal	PA4500	>		probable binding protein component of ABC transporter	Transport of small molecules
as192	5049739	5049879	141	start-span	PA4512	>	*lpxO1*	lipopolysaccharide biosynthetic protein LpxO1	Putative enzymes; Cell wall / LPS / capsule
as193	5055080	5055129	50	internal	PA4514	<		probable outer membrane receptor for iron transport	Transport of small molecules
as194	5055132	5055185	54	internal	PA4514	<		probable outer membrane receptor for iron transport	Transport of small molecules
as195	5071575	5071963	389	internal	PA4527	>	*pilC*	still frameshift type 4 fimbrial biogenesis protein PilC	Motility & Attachment

as196	5071970	5072154	185	internal	PA4527	>	*pilC*	still frameshift type 4 fimbrial biogenesis protein PilC	Motility & Attachment
as197	5101107	5101200	94	internal	PA4554	>	*pilY1*	type 4 fimbrial biogenesis protein PilY1	Motility & Attachment
as198	5101511	5101611	101	internal	PA4554	>	*pilY1*	type 4 fimbrial biogenesis protein PilY1	Motility & Attachment
as199	5141781	5141969	189	end-span	PA4591	<		hypothetical protein	Hypothetical, unclassified, unknown
as200	5148538	5148668	131	start-span	PA4595	<		probable ATP-binding component of ABC transporter	Transport of small molecules
as201	5274032	5274193	162	internal	PA4696	<	*ilvI*	acetolactate synthase large subunit	Biosynthesis of cofactors, prosthetic groups and carriers; Amino acid biosynthesis and metabolism
as202	5296232	5296293	62	internal	PA4717	>		conserved hypothetical protein	Hypothetical, unclassified, unknown
as203	5296311	5296538	228	internal	PA4717	>		conserved hypothetical protein	Hypothetical, unclassified, unknown
as204	5301771	5301838	68	internal	PA4722	<		probable aminotransferase	Putative enzymes
as205	5356355	5356495	141	internal	PA4770	>	*lldP*	L-lactate permease	Transport of small molecules
as206	5375454	5375529	76	end-span	PA4786	>		probable short-chain dehydrogenase	Putative enzymes
as207	5380813	5380919	107	internal	PA4793	>		hypothetical protein	Hypothetical, unclassified, unknown
as208	5382370	5382497	128	end-span	PA4796	>		hypothetical protein	Hypothetical, unclassified, unknown
as209	5408036	5408137	102	internal	PA4818	<		conserved hypothetical protein	Membrane proteins
as210	5473394	5473504	111	internal	PA4877	<		hypothetical protein	Hypothetical, unclassified, unknown
as211	5522339	5522410	72	start-span	PA4923	>		conserved hypothetical protein	Hypothetical, unclassified, unknown
as212	5532791	5532916	126	internal	PA4929	>		hypothetical protein	Membrane proteins
as214	5545002	5545131	130	internal	PA4941	<	*hflC*	protease subunit HflC	Translation, post-translational modification, degradation; Cell division
as216	5580862	5581109	248	start-span	PA4972	>		hypothetical protein	Hypothetical, unclassified, unknown
as217	5584935	5585163	229	internal	PA4974	>		probable outer membrane protein precursor	Protein secretion/export apparatus
as218	5585580	5585652	73	end-span	PA4975	<		NAD(P)H quinone oxidoreductase	Energy metabolism
as219	5602759	5602870	112	internal	PA4986	>		probable oxidoreductase	Putative enzymes
as220	5617657	5617793	137	internal	PA5000	<	*wapR*	alpha-1,3-rhamnosyltransferase WapR	Putative enzymes; Cell wall / LPS / capsule
as221	5618584	5618634	51	internal	PA5001	<		hypothetical protein	Hypothetical, unclassified, unknown
as222	5655041	5655112	72	internal	PA5024	>		conserved hypothetical protein	Hypothetical, unclassified, unknown
as223	5678557	5678652	96	internal	PA5042	<	*pilO*	type 4 fimbrial biogenesis protein PilO	Motility & Attachment
asponA****	5680819	5681167	349	start-span	PA5045	>	*ponA*	penicillin-binding protein 1A	Cell wall / LPS / capsule
as225	5684803	5684910	108	end-span	PA5047	<		hypothetical protein	Hypothetical, unclassified, unknown
as226	5699012	5699062	51	internal	PA5058	>	*phaC2*	poly(3-hydroxyalkanoic acid) synthase 2	Central intermediary metabolism
as228	5725557	5725795	239	internal	PA5087	<		hypothetical protein	Hypothetical, unclassified, unknown
as229	5739066	5739203	138	internal	PA5097	<		probable amino acid permease	Membrane proteins; Transport of small molecules
as230	5785777	5785905	129	internal	PA5136	>		hypothetical protein	Hypothetical, unclassified, unknown
as231	5799079	5799208	130	internal	PA5150	<		probable short-chain dehydrogenase	Putative enzymes
as232	5817430	5817493	64	internal	PA5167	>		probable c4-dicarboxylate-binding protein	Membrane proteins; Transport of small molecules
as233	5824524	5824599	76	internal	PA5172	>	*arcB*	ornithine carbamoyltransferase, catabolic	Amino acid biosynthesis and metabolism
as234	5844451	5844527	77	end-span	PA5192	<	*pckA*	phosphoenolpyruvate carboxykinase	Carbon compound catabolism; Energy metabolism
as235	5863452	5863719	268	start-span	PA5208	<		conserved hypothetical protein	Hypothetical, unclassified, unknown
as236	5868209	5868408	200	internal	PA5213	<	*gcvP1*	glycine cleavage system protein P1	Central intermediary metabolism; Amino acid biosynthesis and metabolism
as237	5896925	5897003	79	internal	PA5238	<		probable O-antigen acetylase	Putative enzymes; Membrane proteins; Cell wall / LPS / capsule
as238	5897008	5897057	50	internal	PA5238	<		probable O-antigen acetylase	Putative enzymes; Membrane proteins; Cell wall / LPS / capsule
as239	5926819	5926963	145	internal	PA5264	<		hypothetical protein	Hypothetical, unclassified, unknown; Membrane proteins
as240	6059529	6059600	72	internal	PA5380	>	*gbdR*	GbdR	Transcriptional regulators
as241	6090655	6090800	146	start-span	PA5412	<		hypothetical protein	Hypothetical, unclassified, unknown
as242	6135711	6135789	79	internal	PA5446	>		hypothetical protein	Hypothetical, unclassified, unknown
as243	6141133	6141239	107	internal	PA5451	<	*wzm*	membrane subunit of A-band LPS efflux transporter	Cell wall / LPS / capsule; Membrane proteins; Transport of small molecules
as244	6157855	6157964	110	internal	PA5468	>		probable citrate transporter	Transport of small molecules
as245	6158032	6158203	172	end-span	PA5468	>		probable citrate transporter	Transport of small molecules
as245	6158032	6158203	172	start-span	PA5469	>		conserved hypothetical protein	Membrane proteins
as246	6171684	6171915	232	start-span	PA5480	>		hypothetical protein	Hypothetical, unclassified, unknown
as247	6183216	6183389	174	internal	PA5492	>		conserved hypothetical protein	Hypothetical, unclassified, unknown
as248	6218749	6219078	330	end-span	PA5525	>		probable transcriptional regulator	Transcriptional regulators
as249	6245830	6245973	144	internal	PA5551	<		hypothetical protein	Hypothetical, unclassified, unknown
as250	6247796	6247909	114	end-span	PA5553	<	*atpC*	ATP synthase epsilon chain	Energy metabolism

* Coordinates and length of the sRNAs predicted by RNA-Seq data.

**The antisense sRNAs were categorize depending on their position with respect to the opposite gene as “start-span” (the asRNA spans over the start of the antisense gene), “internal” (the asRNA start and end within the antisense gene), or “end-span” (the asRNA spans over the end of the gene).

***Some asRNAs overlap with 2 genes.

****Coordinates validated by 5´and 3´-RACE experiments.
