# Supplementary material for: Expression of antisense small RNAs in response to stress in Pseudomonas aeruginosa
Source: BMC Genomics. 2014 Sep 11;15(1):783. doi: 10.1186/1471-2164-15-783 (PMC4180829; doi:10.1186/1471-2164-15-783)

Additional file 3. Antisense transcription in the *wbp* operon. Genes are depicted in blue and asRNAs in green.

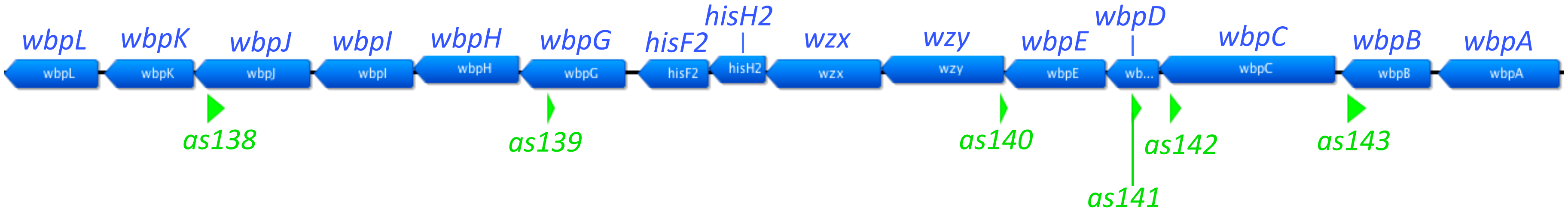

Supplement: Supplementary file 3 — Additional file 3: Antisense transcription in the wbp operon. (PDF 74 KB) [file 12864_2014_6485_MOESM3_ESM.pdf]
