# Supplementary material for: Expression of antisense small RNAs in response to stress in Pseudomonas aeruginosa
Source: BMC Genomics. 2014 Sep 11;15(1):783. doi: 10.1186/1471-2164-15-783 (PMC4180829; doi:10.1186/1471-2164-15-783)
Supplement: Supplementary file 4 — Additional file 4: Antisense sRNAs differentially expressed. (PDF 83 KB) [file 12864_2014_6485_MOESM4_ESM.pdf]

Additional file 4. Antisense sRNAs differentially expressed.

Name	Start*	End*	Length*	Direction asRNA	Fold change**
**asRNAs differentially expressed during AZI (10xMIC) treatment**					
as165	4242856	4242966	111	<	0.12
as210	5473394	5473504	111	>	0.21
as94	2027457	2027571	115	<	0.26
as230	5785777	5785905	129	<	0.32
as77	1640773	1640948	176	>	0.38
as100	2128729	2128826	98	<	0.39
as42	977508	977658	151	>	0.42
as183	4781848	4781947	100	<	2.55
as99	2121071	2121240	170	>	2.69
as123	3015850	3015969	120	<	2.90
as231	5799079	5799208	130	>	2.94
as115	2902292	2902391	100	<	3.30
as111	2756364	2756944	581	<	3.34
as117	2946138	2946205	68	<	4.87
as244	6157855	6157964	110	<	11.33
as184	4842427	4842583	157	<	27.49
**asRNAs differentially expressed during AZT (3xMIC) treatment**					
as6	182516	182617	102	<	0.09
as48	1060351	1060676	326	>	0.26
as104	2445134	2445251	118	>	0.32
as19	448929	449284	356	>	0.33
as119	2959629	2959767	139	<	0.36
as78	1698424	1698534	111	>	0.41
as120	2982707	2982848	142	<	2.06
as29	701233	701489	257	<	2.07
as163	4166790	4167046	257	<	2.20
as10	288493	288639	147	>	2.30
as200	5148538	5148668	131	>	2.33
as228	5725557	5725795	239	>	2.34
as195	5071575	5071963	389	<	2.51
asdnaA****	1320	1784	465	<	2.79
as146	3612043	3612282	240	>	2.82
asponA****	5680819	5681167	349	<	2.94
as205	5356355	5356495	141	<	3.13
as162	4156819	4156946	128	<	3.24
as45	1027896	1028083	188	<	3.56
as135	3414435	3414491	57	<	4.21
as181	4773392	4773503	112	>	5.50
as221	5618584	5618634	51	>	6.08
as244	6157855	6157964	110	<	6.37
as32	749460	749562	103	<	6.84
as39	921673	921840	168	>	20.82
as152	3866017	3866206	190	<	21.77
as128	3119347	3119453	107	>	22.06
**asRNAs differentially expressed during CEF (3xMIC) treatment**					
as6	182516	182617	102	<	0.08
as75	1588434	1588549	116	<	0.09
as210	5473394	5473504	111	>	0.12
as48	1060351	1060676	326	>	0.14
as83	1838134	1838246	113	<	0.16

as119	2959629	2959767	139	<	0.17
as19	448929	449284	356	>	0.20
as43	990194	990309	116	<	0.35
as138	3530607	3530794	188	>	0.37
as94	2027457	2027571	115	<	0.39
as235	5863452	5863719	268	>	0.43
as146	3612043	3612282	240	>	2.10
as29	701233	701489	257	<	2.17
as24	646779	646892	114	>	2.21
as200	5148538	5148668	131	>	2.22
as45	1027896	1028083	188	<	2.32
as192	5049739	5049879	141	<	2.45
as4	35866	35974	109	>	4.02
as16	353325	353458	134	>	4.93
as145	3593054	3593188	135	<	7.72
as129	3120806	3120915	110	<	12.58
as39	921673	921840	168	>	24.03
as56	1141250	1141364	115	<	32.30
**asRNAs differentially expressed during CIP (3xMIC) treatment**					
as6	182516	182617	102	<	0.02
as92	2012282	2012454	173	>	0.05
as64	1484863	1485037	175	<	0.07
as169	4329401	4329645	245	>	0.07
as104	2445134	2445251	118	>	0.10
as157	4048252	4048377	126	>	0.11
as183	4781848	4781947	100	<	0.11
as131	3173549	3173835	287	<	0.13
as110	2756154	2756267	114	<	0.15
as23	557312	557569	258	>	0.17
as111	2756364	2756944	581	<	0.19
as231	5799079	5799208	130	>	0.20
as29	701233	701489	257	<	0.20
as21	544658	544778	121	<	0.21
as77	1640773	1640948	176	>	0.23
as19	448929	449284	356	>	0.23
as225	5684803	5684910	108	>	0.24
as165	4242856	4242966	111	<	0.24
as31	748895	749047	153	<	0.27
as12	291428	291543	116	>	0.31
as101	2314771	2314932	162	<	0.34
as48	1060351	1060676	326	>	0.34
as55	1135776	1135875	100	<	0.36
as38	898637	898918	282	>	0.37
as43	990194	990309	116	<	0.38
as94	2027457	2027571	115	<	0.40
as98	2120607	2120921	315	>	0.43
as138	3530607	3530794	188	>	0.47
as235	5863452	5863719	268	>	0.49
as9	263722	263909	188	<	0.49
as100	2128729	2128826	98	<	2.05
asponA****	5680819	5681167	349	<	2.06
as179	4763739	4763790	52	>	2.22
as208	5382370	5382497	128	<	2.48
as52	1098349	1098557	209	>	2.80
as26	659114	659270	157	>	2.84
as160	4092478	4092590	113	>	2.97

as163	4166790	4167046	257	<	3.05
as7	184720	184861	142	<	3.26
as24	646779	646892	114	>	3.35
as45	1027896	1028083	188	<	3.54
as181	4773392	4773503	112	>	3.69
as32	749460	749562	103	<	3.77
as115	2902292	2902391	100	<	4.45
asdnaA****	1320	1784	465	<	4.67
as136	3508613	3508673	61	<	6.06
as4	35866	35974	109	>	6.41
as152	3866017	3866206	190	<	6.96
as16	353325	353458	134	>	12.17
as128	3119347	3119453	107	>	16.61
as122	3013744	3013890	147	>	22.25
as129	3120806	3120915	110	<	24.88
**asRNAs differentially expressed during CO (3xMIC) treatment**					
as6	182516	182617	102	<	0.02
as37	817964	818095	132	<	0.10
as19	448929	449284	356	>	0.11
as230	5785777	5785905	129	<	0.12
as201	5274032	5274193	162	>	0.14
as131	3173549	3173835	287	<	0.14
as64	1484863	1485037	175	<	0.18
as104	2445134	2445251	118	>	0.18
as235	5863452	5863719	268	>	0.19
as48	1060351	1060676	326	>	0.20
as83	1838134	1838246	113	<	0.20
as92	2012282	2012454	173	>	0.20
as12	291428	291543	116	>	0.21
as206	5375454	5375529	76	<	0.30
as9	263722	263909	188	<	0.30
as203	5296311	5296538	228	<	0.32
as42	977508	977658	151	>	0.32
asponA****	5680819	5681167	349	<	0.32
as225	5684803	5684910	108	>	0.33
as11	290529	290665	137	>	0.34
as55	1135776	1135875	100	<	0.34
as44	1026460	1026563	104	>	0.36
as169	4329401	4329645	245	>	0.39
as82	1790530	1790650	121	>	0.42
as81	1785747	1785881	135	>	0.45
as146	3612043	3612282	240	>	2.25
as119	2959629	2959767	139	<	2.30
as192	5049739	5049879	141	<	2.37
as90	1954335	1954415	81	<	2.72
as120	2982707	2982848	142	<	2.95
as78	1698424	1698534	111	>	3.04
as16	353325	353458	134	>	3.41
as221	5618584	5618634	51	>	3.87
as247	6183216	6183389	174	<	4.48
as205	5356355	5356495	141	<	4.96
as162	4156819	4156946	128	<	5.35
as57	1207482	1207531	50	<	5.44
as121	3005381	3005497	117	<	6.22
as25	654775	654912	138	>	6.25
as36	798624	798854	231	>	6.86

as20	521726	521838	113	>	36.01
**asRNAs differentially expressed during H2O2 (1 mM) treatment**					
as6	182516	182617	102	<	0.06
as48	1060351	1060676	326	>	0.35
as104	2445134	2445251	118	>	0.45
as19	448929	449284	356	>	0.45
as84	1911985	1912118	134	>	2.05
asdnaA****	1320	1784	465	<	2.11
as29	701233	701489	257	<	2.28
as146	3612043	3612282	240	>	2.96
as174	4666087	4666202	116	<	3.31
as205	5356355	5356495	141	<	3.89
as143	3542803	3542997	195	>	4.70
as244	6157855	6157964	110	<	5.40
as120	2982707	2982848	142	<	5.79
as221	5618584	5618634	51	>	6.24
as145	3593054	3593188	135	<	6.51
as20	521726	521838	113	>	7.47
as39	921673	921840	168	>	9.95
**asRNAs differentially expressed during MP (3xMIC) treatment**					
as6	182516	182617	102	<	0.05
as19	448929	449284	356	>	0.09
as201	5274032	5274193	162	>	0.09
as77	1640773	1640948	176	>	0.09
as104	2445134	2445251	118	>	0.10
as235	5863452	5863719	268	>	0.14
as12	291428	291543	116	>	0.15
as131	3173549	3173835	287	<	0.16
as48	1060351	1060676	326	>	0.16
as55	1135776	1135875	100	<	0.19
as230	5785777	5785905	129	<	0.22
as138	3530607	3530794	188	>	0.26
as169	4329401	4329645	245	>	0.27
as225	5684803	5684910	108	>	0.31
as220	5617657	5617793	137	>	0.33
as203	5296311	5296538	228	<	0.41
as31	748895	749047	153	<	0.44
as94	2027457	2027571	115	<	0.45
as23	557312	557569	258	>	0.45
asponA****	5680819	5681167	349	<	0.48
as81	1785747	1785881	135	>	2.20
as146	3612043	3612282	240	>	2.62
as200	5148538	5148668	131	>	2.82
as16	353325	353458	134	>	3.57
as163	4166790	4167046	257	<	3.92
as248	6218749	6219078	330	<	4.08
as29	701233	701489	257	<	4.51
as162	4156819	4156946	128	<	5.46
as62	1455941	1456046	106	>	15.67
as236	5868209	5868408	200	>	21.07
**asRNAs differentially expressed during NaCl (0.5 M) treatment**					
as6	182516	182617	102	<	0.12
as12	291428	291543	116	>	0.16
as19	448929	449284	356	>	0.20
as48	1060351	1060676	326	>	0.27
as55	1135776	1135875	100	<	0.33

as235	5863452	5863719	268	>	0.36
as74	1558500	1558752	253	<	0.41
as84	1911985	1912118	134	>	2.55
as214	5545002	5545131	130	>	2.71
as36	798624	798854	231	>	3.58
as236	5868209	5868408	200	>	4.13
as204	5301771	5301838	68	>	6.66
as62	1455941	1456046	106	>	12.37
**asRNAs differentially expressed during PP (3xMIC) treatment**					
as6	182516	182617	102	<	0.07
as104	2445134	2445251	118	>	0.09
as48	1060351	1060676	326	>	0.25
as94	2027457	2027571	115	<	0.29
as19	448929	449284	356	>	0.34
as138	3530607	3530794	188	>	0.39
as43	990194	990309	116	<	0.40
as92	2012282	2012454	173	>	0.42
as55	1135776	1135875	100	<	0.50
as212	5532791	5532916	126	<	2.11
as181	4773392	4773503	112	>	2.31
as44	1026460	1026563	104	>	2.36
as162	4156819	4156946	128	<	2.47
as96	2113618	2113734	117	<	2.71
as143	3542803	3542997	195	>	2.79
asponA****	5680819	5681167	349	<	2.83
as146	3612043	3612282	240	>	2.95
as91	1954420	1954469	50	<	3.14
as221	5618584	5618634	51	>	4.17
as205	5356355	5356495	141	<	4.22
as16	353325	353458	134	>	4.25
as244	6157855	6157964	110	<	6.49
as129	3120806	3120915	110	<	21.17
**asRNAs differentially expressed during TET (3xMIC) treatment**					
as92	2012282	2012454	173	>	0.02
as78	1698424	1698534	111	>	0.04
as19	448929	449284	356	>	0.05
as165	4242856	4242966	111	<	0.07
as21	544658	544778	121	<	0.08
as12	291428	291543	116	>	0.10
as6	182516	182617	102	<	0.13
as26	659114	659270	157	>	0.14
as217	5584935	5585163	229	<	0.15
as94	2027457	2027571	115	<	0.16
asponA****	5680819	5681167	349	<	0.18
as31	748895	749047	153	<	0.20
as120	2982707	2982848	142	<	0.24
as42	977508	977658	151	>	0.25
as157	4048252	4048377	126	>	0.26
as53	1119615	1119886	272	>	0.26
as29	701233	701489	257	<	0.28
as9	263722	263909	188	<	0.31
as23	557312	557569	258	>	0.33
as192	5049739	5049879	141	<	0.36
as250	6247796	6247909	114	>	0.40
as144	3592018	3592128	111	>	0.43
as206	5375454	5375529	76	<	0.47

as67	1499690	1499742	53	<	0.49
as74	1558500	1558752	253	<	2.30
as60	1394817	1395002	186	>	2.40
as11	290529	290665	137	>	2.43
as154	3944227	3944342	116	<	2.98
as111	2756364	2756944	581	<	3.03
as162	4156819	4156946	128	<	3.51
as104	2445134	2445251	118	>	3.72
as163	4166790	4167046	257	<	4.18
as239	5926819	5926963	145	>	4.37
as142	3540908	3541027	120	>	4.83
as203	5296311	5296538	228	<	5.44
as54	1135102	1135286	185	<	5.69
as236	5868209	5868408	200	>	6.63
as241	6090655	6090800	146	>	6.76
as30	718785	718934	150	>	6.97
as136	3508613	3508673	61	<	7.51
as210	5473394	5473504	111	>	7.66
as99	2121071	2121240	170	>	7.79
as107	2623295	2623503	209	<	9.26
as25	654775	654912	138	>	10.98
as16	353325	353458	134	>	11.47
as64	1484863	1485037	175	<	11.74
as216	5580862	5581109	248	<	12.15
as244	6157855	6157964	110	<	14.57
as167	4327946	4328181	236	>	16.07
as88	1949036	1949142	107	>	19.62
as117	2946138	2946205	68	<	20.08
as49	1066483	1066610	128	>	20.16
as20	521726	521838	113	>	21.57
as76	1639661	1639805	145	>	24.33
as50	1073865	1074007	143	<	25.41
as134	3395484	3395684	201	>	26.97
as47	1056103	1056248	146	<	27.11
as115	2902292	2902391	100	<	42.28
as247	6183216	6183389	174	<	43.19

asRNAs differentially expressed during TOB (3xMIC) treatment

as92	2012282	2012454	173	>	0.07
as165	4242856	4242966	111	<	0.08
as42	977508	977658	151	>	0.11
as120	2982707	2982848	142	<	0.13
as6	182516	182617	102	<	0.14
as78	1698424	1698534	111	>	0.16
as19	448929	449284	356	>	0.17
asponA****	5680819	5681167	349	<	0.18
as119	2959629	2959767	139	<	0.21
as74	1558500	1558752	253	<	0.25
as12	291428	291543	116	>	0.36
as26	659114	659270	157	>	0.40
as206	5375454	5375529	76	<	0.40
as183	4781848	4781947	100	<	0.41
as21	544658	544778	121	<	0.45
as200	5148538	5148668	131	>	0.47
as31	748895	749047	153	<	0.48
as7	184720	184861	142	<	2.07
as214	5545002	5545131	130	>	2.31

as77	1640773	1640948	176	>	2.36
as195	5071575	5071963	389	<	2.40
as250	6247796	6247909	114	>	2.43
as39	921673	921840	168	>	2.46
as38	898637	898918	282	>	2.53
as146	3612043	3612282	240	>	2.55
as168	4328357	4328518	162	>	2.67
as203	5296311	5296538	228	<	2.79
as179	4763739	4763790	52	>	2.94
as176	4759525	4759686	162	>	3.38
as233	5824524	5824599	76	<	3.67
as107	2623295	2623503	209	<	3.75
as182	4780416	4780494	79	>	3.80
as247	6183216	6183389	174	<	3.82
as97	2113817	2113927	111	<	3.94
as115	2902292	2902391	100	<	4.15
as52	1098349	1098557	209	>	4.22
as45	1027896	1028083	188	<	4.34
as27	680736	680853	118	<	4.41
as50	1073865	1074007	143	<	4.57
as76	1639661	1639805	145	>	4.60
as95	2113335	2113442	108	<	5.62
as25	654775	654912	138	>	6.19
as138	3530607	3530794	188	>	6.37
as64	1484863	1485037	175	<	6.41
as99	2121071	2121240	170	>	7.55
as110	2756154	2756267	114	<	7.72
as62	1455941	1456046	106	>	7.76
as30	718785	718934	150	>	8.27
as236	5868209	5868408	200	>	9.24
as96	2113618	2113734	117	<	11.00
as205	5356355	5356495	141	<	11.76
as93	2027111	2027193	83	<	14.45
as162	4156819	4156946	128	<	14.83
as16	353325	353458	134	>	18.51
as71	1504553	1504676	124	<	20.05
as245	6158032	6158203	172	<	20.79
as70	1503843	1503937	95	<	30.29
as244	6157855	6157964	110	<	37.70
as136	3508613	3508673	61	<	41.81
as20	521726	521838	113	>	51.70
as36	798624	798854	231	>	104.80

asRNAs differentially expressed during early stationary phase

as6	182516	182617	102	<	0.02
as231	5799079	5799208	130	>	0.11
as78	1698424	1698534	111	>	0.05
as201	5274032	5274193	162	>	0.05
as75	1588434	1588549	116	<	0.10
as161	4110202	4110321	120	>	0.11
as190	5018832	5019000	169	>	0.11
as44	1026460	1026563	104	>	0.13
as121	3005381	3005497	117	<	0.15
as230	5785777	5785905	129	<	0.16
as212	5532791	5532916	126	<	0.18
as203	5296311	5296538	228	<	0.19
as82	1790530	1790650	121	>	0.20

as48	1060351	1060676	326	>	0.22
as26	659114	659270	157	>	0.24
as19	448929	449284	356	>	0.26
as7	184720	184861	142	<	0.27
as104	2445134	2445251	118	>	0.30
as43	990194	990309	116	<	0.31
as165	4242856	4242966	111	<	0.32
as216	5580862	5581109	248	<	0.32
as210	5473394	5473504	111	>	0.32
as23	557312	557569	258	>	0.32
as74	1558500	1558752	253	<	0.35
as81	1785747	1785881	135	>	0.36
as21	544658	544778	121	<	0.36
as42	977508	977658	151	>	0.37
as138	3530607	3530794	188	>	0.44
as235	5863452	5863719	268	>	0.47
as144	3592018	3592128	111	>	0.48
as55	1135776	1135875	100	<	0.49
as94	2027457	2027571	115	<	0.49
as9	263722	263909	188	<	0.56
as53	1119615	1119886	272	>	0.57
as92	2012282	2012454	173	>	0.70
as31	748895	749047	153	<	0.90
as60	1394817	1395002	186	>	1.51
as29	701233	701489	257	<	1.70
as67	1499690	1499742	53	<	1.83
as214	5545002	5545131	130	>	1.89
as142	3540908	3541027	120	>	2.00
as143	3542803	3542997	195	>	2.09
as200	5148538	5148668	131	>	2.39
as111	2756364	2756944	581	<	2.77
as38	898637	898918	282	>	2.91
as84	1911985	1912118	134	>	3.18
as68	1502841	1502987	147	<	3.27
as204	5301771	5301838	68	>	3.64
as110	2756154	2756267	114	<	3.67
as228	5725557	5725795	239	>	3.91
as168	4328357	4328518	162	>	3.91
as157	4048252	4048377	126	>	4.10
as101	2314771	2314932	162	<	4.12
as194	5055132	5055185	54	>	4.31
as158	4070600	4070735	136	>	4.60
as169	4329401	4329645	245	>	5.21
as197	5101107	5101200	94	<	5.35
as249	6245830	6245973	144	>	7.19
as99	2121071	2121240	170	>	7.48
as135	3414435	3414491	57	<	7.76
as173	4619821	4619903	83	<	7.97
asdnaA****	1656	1813	158	<	8.70
as62	1455941	1456046	106	>	9.45
as238	5897008	5897057	50	>	11.15
as239	5926819	5926963	145	>	11.43
as195	5071575	5071963	389	<	12.18
as218	5585580	5585652	73	>	12.18
as148	3626660	3626776	117	<	12.90
as147	3625886	3625995	110	<	14.45

as221	5618584	5618634	51	>	14.92
as11	290529	290665	137	>	15.87
as182	4780416	4780494	79	>	16.64
as176	4759525	4759686	162	>	17.15
as34	784213	784303	91	<	18.57
as8	226915	226976	62	<	18.59
as151	3752069	3752155	87	>	20.20
as105	2550592	2550780	189	<	20.43
as196	5071970	5072154	185	<	21.03
as141	3540497	3540593	97	>	21.54
as232	5817430	5817493	64	<	22.70
as133	3357890	3357996	107	>	23.80
as186	4925623	4925733	111	>	23.83
as114	2818927	2819159	233	>	24.56
as149	3631510	3631629	120	<	25.52
as89	1953270	1953371	102	<	28.32
as87	1916046	1916172	127	<	28.79
as178	4761521	4761592	72	>	29.29
as223	5678557	5678652	96	>	30.13
as199	5141781	5141969	189	>	34.59
as237	5896925	5897003	79	>	39.97
as145	3593054	3593188	135	<	42.93
as242	6135711	6135789	79	<	45.32
as13	299094	299181	88	>	57.43
as56	1141250	1141364	115	<	66.44
as137	3517655	3517722	68	>	67.12
as229	5739066	5739203	138	>	72.49
as108	2701170	2701296	127	<	61.53
as122	3013744	3013890	147	>	55.57
as28	697609	697703	95	<	77.29
as164	4185534	4185601	68	<	70.65
as189	5002347	5002404	58	<	71.99
as226	5699012	5699062	51	<	77.00
as59	1323807	1323918	112	>	70.06
as159	4087208	4087269	62	>	73.52
as222	5655041	5655112	72	<	79.82
as172	4616918	4616968	51	<	92.70

* Coordinates and length of the sRNAs predicted by RNA-Seq data.

** The fold change is calculated as the ratio of the intensities between the treated condition and the untreated control. Thus a fold change > 1 indicates that the sRNA is significantly more expressed in the treated condition.

****Coordinates validated by 5´and 3´-RACE experiments.
