# Supplementary material for: Expression of antisense small RNAs in response to stress in Pseudomonas aeruginosa
Source: BMC Genomics. 2014 Sep 11;15(1):783. doi: 10.1186/1471-2164-15-783 (PMC4180829; doi:10.1186/1471-2164-15-783)

Additional file 5. AsponA in *P. aeruginosa* PAO1. AsponA is encoded opposite the beginning of gene *ponA*, encoding the penicillin-binding protein 1A, and overlaps with the predicted -10 and -35 σ^{70} promoter elements. Its coordinates have been validated by 5´- and 3´-RACE. Genes are depicted in blue and asRNAs in green.

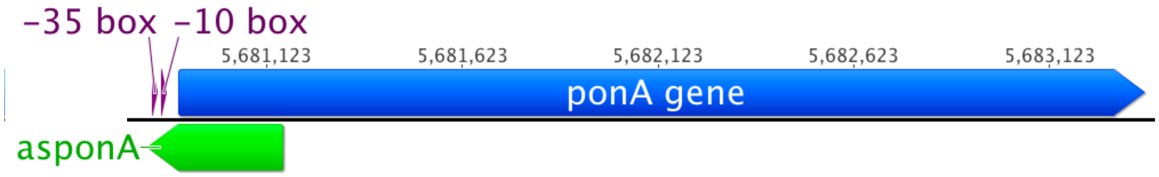

Supplement: Supplementary file 5 — Additional file 5: AsponA in P. aeruginosa PAO1. (PDF 104 KB) [file 12864_2014_6485_MOESM5_ESM.pdf]
