# Supplementary material for: Expression of antisense small RNAs in response to stress in Pseudomonas aeruginosa
Source: BMC Genomics. 2014 Sep 11;15(1):783. doi: 10.1186/1471-2164-15-783 (PMC4180829; doi:10.1186/1471-2164-15-783)
Supplement: Supplementary file 6 — Additional file 6: Primers and adapters used in the study. (PDF 72 KB) [file 12864_2014_6485_MOESM6_ESM.pdf]

Additional file 6. Primers and adapters used in the study.

Primer	Sequence (5´->3´)	Use
23S-1954	AAAAAAAAAAAAAAAAAAACTTACCCGACAAGGAATTTCGC	Removal of 23S rRNA (provided in MICROBExpress kit, Ambion)
23S-2511	AAAAAAAAAAAAAAAAAAGAGCCGACATCGAGGTGCCAAAC	Removal of 23S rRNA (provided in MICROBExpress kit, Ambion)
16S-807	AAAAAAAAAAAAAAAAAATGGACTACCAGGGTATCTAATCC	Removal of 16S rRNA (provided in MICROBExpress kit, Ambion)
16S-1114	AAAAAAAAAAAAAAAAAAGGGTTGCGCTCGTTACGGGACTT	Removal of 16S rRNA (provided in MICROBExpress kit, Ambion)
5S	AAAAAAAAAAAAAAAAAAGCGTTTCACTTCTGAGTTCGGCA	Removal of 5S rRNA
5´RNA adapter	GCUGAUGGCGAUGAAUGAACACUGCGUUUGCUGGCUUUGAUGAAA	5´-RACE
5´RNA adapter-specific primer	GCTGATGGCGATGAATGAACACTG	5´-RACE
5´-GSP1_asponA	AGATGCGCCGGACGCCGATC	5´-RACE *asponA*
5´-GSP2_asponA	CTTCGCCGACATCCCGCAGGA	5´-RACE *asponA*
3´RNA adapter E1	UUCACUGUUCUUAGCGGCCGCAUGCUC-idT	3´-RACE
3´RNA adapter E1 primer	CATGCGGCCGCTAAGAACAGTGA	3´-RACE
3´-GSP1_asponA	GAAATTGTCGTCCTCGGCGGACA	3´-RACE *asponA*
3´-GSP2_asponA	CAGGGCATGGATGAAGTCCTG	3´-RACE *asponA*
