# Supplementary material for: Expression of antisense small RNAs in response to stress in Pseudomonas aeruginosa
Source: BMC Genomics. 2014 Sep 11;15(1):783. doi: 10.1186/1471-2164-15-783 (PMC4180829; doi:10.1186/1471-2164-15-783)
Supplement: Supplementary file 7 — Additional file 7: Script for extracting antisense reads. (PDF 136 KB) [file 12864_2014_6485_MOESM7_ESM.pdf]

Additional file 7. Script for extracting antisense reads.

As *cis*-encoded sRNAs are transcribed from the DNA strand opposite the target RNA, they can only be directly identified in strand-specific libraries, and the analysis should be constrained to reads being in the opposite direction to the genes onto which they align. Therefore, we wish to extract all the reads that are in the reverse direction of the genes. This is done by the script **extract_reverse_intragenic_RNA.sh.** The extracted reads are placed into a new SAM-formatted file with a **.rev_extract** extension. This file only contains reverse intragenic reads. More information about this is available in Gómez-Lozano *et al* (2014).

The script needs three inputs:

```
sh extract_reverse_intragenic_RNA.sh [samfile] [infofile] [buffer]
```

The *SAM file* is the one containing all the sequenced reads.

The file *infofile* has 6 columns, giving information about all the annotated genes:

Column 1: Locus name (PA0001, PA0002…)
Column2: Start
Column 3: End
Column 4: Direction (*first* or *complement*)
Column 5: Gene name (*dnaA*, *dnaN*…)
Column 6: Gene annotation/description

The *buffer* should be set as the length of the reads.

References

Gómez-Lozano, M., Marvig, R. L., Molin, S., & Long, K. S. (2014). Identification of Bacterial Small RNAs by RNA Sequencing. In *Pseudomonas Methods and Protocols* (Vol. 1149, pp. 433–456). New York, NY: Springer Science+Business Media New York. doi:10.1007/978-1-4939-0473-0_34

SCRIPT *extract_reverse_intragenic_RNA.sh*

```
#!/bin/bash
# Rasmus Marvig rn@bio.dtu.dk July 5th 2012
# NB: Script was modified to make is faster.
# (1) An exit statement in the awk-line was incorporated.
# (2) The SAM-file is sorted and fragmented to be processed in smaller parts.

SAMFILE=$1
INFOFILE=$2
BUF=$3

rm tmpS1

tail -n +4 $SAMFILE | sort -nk 4 > tmpS6


awk '$4>=1-200 && $4<520000' tmpS6 > tmpS_m00
awk '$4>=500000-200 && $4<1020000' tmpS6 > tmpS_m01
awk '$4>=1000000-200 && $4<1520000' tmpS6 > tmpS_m02
awk '$4>=1500000-200 && $4<2020000' tmpS6 > tmpS_m03
awk '$4>=2000000-200 && $4<2520000' tmpS6 > tmpS_m04
awk '$4>=2500000-200 && $4<3020000' tmpS6 > tmpS_m05
awk '$4>=3000000-200 && $4<3520000' tmpS6 > tmpS_m06
awk '$4>=3500000-200 && $4<4020000' tmpS6 > tmpS_m07
awk '$4>=4000000-200 && $4<4520000' tmpS6 > tmpS_m08
awk '$4>=4500000-200 && $4<5020000' tmpS6 > tmpS_m09
awk '$4>=5000000-200 && $4<5520000' tmpS6 > tmpS_m10
awk '$4>=5500000-200 && $4<6020000' tmpS6 > tmpS_m11
awk '$4>=6000000-200 && $4<6520000' tmpS6 > tmpS_m12

awk '$2>=1 && $2<500000' $INFOFILE > tmpS_g00
awk '$2>=500000 && $2<1000000' $INFOFILE > tmpS_g01
awk '$2>=1000000 && $2<1500000' $INFOFILE > tmpS_g02
awk '$2>=1500000 && $2<2000000' $INFOFILE > tmpS_g03
awk '$2>=2000000 && $2<2500000' $INFOFILE > tmpS_g04
awk '$2>=2500000 && $2<3000000' $INFOFILE > tmpS_g05
awk '$2>=3000000 && $2<3500000' $INFOFILE > tmpS_g06
awk '$2>=3500000 && $2<4000000' $INFOFILE > tmpS_g07
awk '$2>=4000000 && $2<4500000' $INFOFILE > tmpS_g08
awk '$2>=4500000 && $2<5000000' $INFOFILE > tmpS_g09
awk '$2>=5000000 && $2<5500000' $INFOFILE > tmpS_g10
awk '$2>=5500000 && $2<6000000' $INFOFILE > tmpS_g11
awk '$2>=6000000 && $2<6500000' $INFOFILE > tmpS_g12

for X in 00 01 02 03 04 05 06 07 08 09 10 11 12
do

while read line
do

NAME=`echo $line | awk '{print $1}'`
POS1=`echo $line | awk '{print $2}'`
POS2=`echo $line | awk '{print $3}'`
ORI=`echo $line | awk '{print $4}'`

echo $NAME

if [ "$ORI" == "first" ]; then
```

```
awk -v pos2=$POS2 '{print;if($4>pos2) exit}' tmpS_m$X | awk -v pos1=$POS1 -v pos2=$POS2 -
v buf=$BUF '$4>=pos1-buf+1 && $4<=pos2' | awk '($2==163 || $2==83)' >> tmpS1
fi


if [ "$ORI" == "complement" ]; then
awk -v pos2=$POS2 '{print;if($4>pos2) exit}' tmpS_m$X | awk -v pos1=$POS1 -v pos2=$POS2 -
v buf=$BUF '$4>=pos1-buf+1 && $4<=pos2' | awk '($2==99 || $2==147)' >> tmpS1
fi

done < tmpS_g$X

done


sort tmpS1 | uniq > tmpS2
# Retrieve the paired mate also
cut -f1 tmpS2 | uniq -c | awk '$1==1' | awk '{print $2}' > tmpS3
fgrep -f tmpS3 $SAMFILE >> tmpS2
sort tmpS2 | uniq > tmpS4

head -n3 $SAMFILE > tmpS5

cat tmpS5 tmpS4 > $SAMFILE.rev_extract
mv $SAMFILE.rev_extract .

rm tmpS*
```
